# NoGOA: predicting noisy GO annotations using evidences and sparse representation

**DOI:** 10.1186/s12859-017-1764-z

**Published:** 2017-07-21

**Authors:** Guoxian Yu, Chang Lu, Jun Wang

**Affiliations:** College of Computer and Information Sciences, Southwest University, Chongqing, China

**Keywords:** Gene ontology, GO annotations, Evidence codes, Sparse representation

## Abstract

**Background:**

Gene Ontology (GO) is a community effort to represent functional features of gene products. GO annotations (GOA) provide functional associations between GO terms and gene products. Due to resources limitation, only a small portion of annotations are manually checked by curators, and the others are electronically inferred. Although quality control techniques have been applied to ensure the quality of annotations, the community consistently report that there are still considerable noisy (or incorrect) annotations. Given the wide application of annotations, however, how to identify noisy annotations is an important but yet seldom studied open problem.

**Results:**

We introduce a novel approach called *NoGOA* to predict noisy annotations. NoGOA applies sparse representation on the gene-term association matrix to reduce the impact of noisy annotations, and takes advantage of sparse representation coefficients to measure the semantic similarity between genes. Secondly, it preliminarily predicts noisy annotations of a gene based on aggregated votes from semantic neighborhood genes of that gene. Next, NoGOA estimates the ratio of noisy annotations for each evidence code based on direct annotations in GOA files archived on different periods, and then weights entries of the association matrix via estimated ratios and propagates weights to ancestors of direct annotations using GO hierarchy. Finally, it integrates evidence-weighted association matrix and aggregated votes to predict noisy annotations. Experiments on archived GOA files of six model species (H. sapiens, A. thaliana, S. cerevisiae, G. gallus, B. Taurus and M. musculus) demonstrate that NoGOA achieves significantly better results than other related methods and removing noisy annotations improves the performance of gene function prediction.

**Conclusions:**

The comparative study justifies the effectiveness of integrating evidence codes with sparse representation for predicting noisy GO annotations. Codes and datasets are available at http://mlda.swu.edu.cn/codes.php?name=NoGOA.

**Electronic supplementary material:**

The online version of this article (doi:10.1186/s12859-017-1764-z) contains supplementary material, which is available to authorized users.

## Background

With the influx of biological data, it is difficult for researchers to collect and search functional knowledge of gene products (including proteins and RNAs), as different databases use different schemas to describe gene functions. To overcome this problem, Gene Ontology Consortium (GOC) collaboratively developed Gene Ontology (GO) [1]. GO has two components: GO and GO annotations (GOA) files. GO uses structured vocabularies to annotate molecular function, biological roles and cellular location of gene products in a taxonomic and species-neutral way. Particularly, GO arranges GO terms into three branches: molecular function (MF), biological process (BP) and cellular component (CC). Each branch organizes terms in a direct acyclic graph to reflect hierarchical structure relationship among them. GOA files store functional annotations of gene products, which associate gene products with GO terms. Each annotation encodes the knowledge that the relevant gene products carry out the biological function described by the associated GO term. Hereinafter, for brevity, we abuse annotations of gene products as annotations of genes.

GO annotations are originally extracted from published experimental data by GO curators. These annotations provide solid, dependable sources for function inference [2], and are also biased by the research interests of biologists [3]. With the development and application of high-throughput technologies, accumulated large volume of biological data enable to computationally predict gene functions. Various computational approaches have been proposed to predict gene function without curator intervention [4, 5]. Manually checking these electronically predicted annotations is low throughput and labor-intensive.

Electronically inferred annotations provide a broad coverage and have a significantly larger taxonomic range than manual ones [6, 7]. On the one hand, since these annotations are not checked by curators, they may have lower reliability than manual ones [8]. On the other hand, curated annotations are restricted by experiment protocols and contexts [3]. Therefore, both inferred and curated annotations include some incorrect annotations [9]. As we known, GO is regularly updated with some terms obsolete or appended as the updated biological knowledge. Similarly, annotations of genes are also updated as the accumulated biological evidences and evolved GO. However, we want to remark that the removed annotations in archived GOA files, from our preliminary investigation, do not solely result from updated GO terms and structure. For example, in an archived (date: May 9th, 2016) GOA file of S. cerevisiae, ‘AAC1’ (ADP/ATP Carrier) was annotated with a GO term ‘GO:0006412’ (translation), but ‘AAC1’ was not annotated with ‘GO:0006412’ in a recently archived (date: September 24th, 2016) GOA file. Further investigation using QuickGO [10] shows this removed annotation is not caused by the change of GO. In fact, annotations in archived GOA files have already underwent several quality control measures to ensure consistency and quality [7]. Gross et al. [11] studied the evolution and (in)stability of GO annotations and found that there were evolution operations for annotations. These instable annotations are not only caused by the changes of gene products or ontology, but also by the incorrect (or inappropriate) annotations. Gross et al. [12] further found that past changes in the GO and GOA are non-uniformly distributed over different branches of the ontology. Gillis et al. [13] also showed instabilities of annotation data and detected that 20% annotations of the genes could not be mapped to themselves after a two year interval. Clarke et al. [14] investigated annotations and structural ontology changes from 2004 to 2012, and found that annotation changes are largely responsible for the changes of enrichment analysis on angiogenesis and the most significant terms. These observations suggest that there are some incorrect annotations in GOA files. Hereinafter, we call these incorrect annotations as *noisy* annotations. These noisy annotations can mislead the downstream analysis and applications, such as GO enrichment analysis [14, 15], diseases analysis [16], drug repositioning [17] and so on.

Some researchers tried to improve annotation quality using association rules. Faria et al. [18] summarized that erroneous annotations, incomplete annotations, and inconsistent annotations affect the annotation quality, and introduced a association rule learning method to evaluate inconsistent annotations in the MF branch. Agapito et al. [19] considered different GO terms have different information contents, and proposed a weighted association rule solution based on the information contents to improve annotation consistencies. This solution only uses one ontology. Agapito et al. [20] extended this solution to mine cross-ontology association rules, i.e., association rules whose terms belong to different branches of GO. Despite these efforts to avoid errors and inconsistencies, most groups are more concerned with replenishing (or predicting) new GO annotations of genes than removing noisy ones [5, 7], and how to predict noisy annotations is a rarely studied but essential problem.

Each GO annotation is tagged with an evidence code, recording the type of evidence (or source) the annotation extracted from [1, 8]. GO currently uses 21 evidence codes and divides them into four categories, which are shown in Table 1. All these evidence codes are reviewed by curators, except IEA (Inferred from Electronic Annotation). There are several studies on assessing GO annotation quality with evidence codes. Thomas et al. [21] recommended to use evidence codes as indicator for the reliability of annotations. They investigated annotations of different species and categorized homology-based, literature-based and other annotations, and found that literature-based (experimental and author statement) annotations are more reliable than others. Clark et al. [22] investigated the quality of NAS (Non-traceable Author Statement) and IEA annotations, and found IEA annotations were much more reliable in MF branch than NAS ones. Gross et al. [11] estimated stability and quality of different evidence codes by considering evolutionary changes. Buza et al. [23] took advantage of GO annotation quality score based on a ranking of evidence codes to assess the quality of annotations available for specific biological processes. Jones et al. [24] found that electronic annotators that using ISS (Inferred from Sequence or structural Similarity) annotations as the basis of predictions are likely to have higher false prediction rates, and suggested to consider avoiding ISS annotations where possible. All these methods just analyze the quality of annotations for different evidence codes. However, none of them pay attention to automatically predicting noisy GO annotations.

**Table 1:** Four categories of evidence codes used in GO and their meanings

Experimental	Computational	Author	Curatorial
EXP: inferred from experiment	ISS: inferred from sequence or structural similarity	TAS: traceable author statement	IC: inferred by curator
IDA: inferred from direct assay	ISO: inferred from sequence orthology	NAS: non-traceable author statement	ND: no biological data available
IPI: inferred from physical interaction	ISA: inferred from sequence alignment		
IMP: inferred from mutant phenotype	ISM: inferred from sequence model		
IGI: inferred from genetic interaction	IGC: inferred from genomic context		
IEP: inferred from expression pattern	IBA: inferred from biological aspect of ancestor		
	IBD: inferred from biological aspect of descendant		
	IKR: inferred from key residues		
	IRD: inferred from rapid divergence		
	RCA: inferred from reviewed computational analysis		
	IEA: inferred from electronic annotation		

Evidence codes are also adopted to measure the semantic similarity between genes [25, 26]. Benabderrahmane et al. [25] assigned different weights to GO annotations based on the evidence codes tagged with these annotations, and used a graph-based similarity measure to compute the semantic similarity between genes. They observed this evidence weighted semantic similarity was more consistent with the sequence similarity between genes than the counterpart without considering the evidence codes. Semantic similarity is found to be positively correlated with the sequence similarity between genes, protein-protein interactions and other types of biological data [27, 28]. Given that, it has been applied to predict the missing annotations of incompletely annotated genes and to validate protein-protein interactions [29–31]. Lu et al. [32] pioneered noisy annotations prediction and suggested a method called NoisyGOA. NoisyGOA firstly computes a vector-based semantic similarity between genes, and a taxonomic similarity between terms using GO hierarchy. Then, it aggregates the maximal taxonomic similarity between terms annotated to a gene and terms annotated to neighborhood genes. After that, it takes terms with the smallest aggregated scores as noisy annotations of the gene. However, NoisyGOA is still suffered from noisy annotations in measuring the semantic similarity between genes, and it does not differentiate the reliability of different annotations.

There are more than 43,000 terms in GO and each gene is often annotated with dozens or several of these terms. From this perspective, the gene-term association matrix, encoding GO annotations of genes, is sparse with some noisy entries. To accurately measure the semantic similarity between genes, we use sparse representation [33], which has been extensively applied in image and signal de-noising, sparse feature learning [34]. When the input signals are sparse with some noises, sparse representation shows superiority in capturing the ground-truth signals. Motivated by these observations, we advocate to integrate sparse representation with evidence codes to predict noisy annotations and introduce an approach called *NoGOA*. NoGOA applies sparse representation on the gene-term matrix to compute the sparse representation coefficients and takes the coefficients as the semantic similarity between genes. Then, it votes noisy annotations of a gene based on annotations of its neighborhood genes. Next, it estimates ratios of noisy annotations for each evidence code based on archived GOA files in different releases, and weights each entry of the gene-term matrix by estimated ratios and GO hierarchy. The final prediction of noisy annotations is obtained from the integration of the weighted gene-term matrix and the aggregated votes from neighborhood genes.

There are no off-the-shelf noisy annotations to quantitatively study the performance of NoGOA in predicting noisy annotations. For this purpose, we collected GOA files archived on four different periods, May 2015, May 2016, September 2015 and September 2016. For each year, we call the GOA file archived in May as the *historical* one, and the GOA file archived in September as the *recent* one. We take the annotations available in the historical GOA file but absent in the recent one as noisy annotations. Based on this protocol, we conducted experiments on archived GOA files of six model species (H. Sapiens, A. thaliana, S. cerevisiae, G. gallus, B. Taurus and M. musculus). Comparative study shows that noisy annotations are predictable and NoGOA outperforms other related techniques in predicting noisy annotations. The empirical study also demonstrates removing noisy annotations can significantly improve the performance of gene function prediction.

## Methods

Let $\mathbf {A} \in \mathbb {R}^{N\times |\mathcal {T}|}$ be a gene-term association matrix, *N* is the number of genes, $\mathcal {T}$ is the set of GO terms and $|\mathcal {T}|$ is the cardinality of $\mathcal {T}$. **A** is defined as follows:

1 $$ \mathbf{A}(i,t)= \left\{ \begin{array}{l} 1, \ \text{if gene}\ i\ \text{is annotated with}\\ \text{term}\ t\ \text{or}\ t^{\prime}\text{s descendants}\\ 0, \ \text{otherwise} \\ \end{array} \right.   $$

The objective of NoGOA is to identify noisy annotations in **A** and update corresponding entries from 1 to 0. Although identifying noisy annotations can be viewed as a different face of gene function prediction, we still would like to remark that identifying noisy annotations is different from replenishing missing annotations of incompletely annotated genes [29, 31], which updates some entries of **A** from 0 to 1. It is also different from negative examples selection [35, 36], which updates some entries of **A** from 0 to -1 and indicates that the relevant genes are clearly not annotated with the given GO terms.

### Preliminary noisy annotations prediction using sparse representation

In this section, we firstly compute the semantic similarity between genes, and then use this similarity to select neighborhood genes of a gene and to preliminarily infer noisy annotations. There are some noisy annotations in the GOA files. In other words, there are some noisy entries in **A**. Although various semantic similarity measures have been proposed and widely applied, most of them are still suffered from shallow and incomplete GO annotations of genes [27, 28, 37, 38]. Sparse representation has been widely and successfully applied to handle images with blurs, speech data with noises and to recover samples with noisy features [33, 34]. Actually, the portion of non-zero entries in **A** is no more than 2%. Therefore **A** is a sparse matrix with some noisy entries. Given the characteristics of **A** and of sparse representation, we resort to sparse representation on **A** to measure the semantic similarity between genes. In this paper, we use an *l*_1_ norm regularized sparse representation objective function as follows:

2 $$ \hat{{\gamma_{i}}}=\arg\underset{{\boldsymbol{\gamma}_{i}}}\min ||\mathbf{A}(i,\cdot)-\boldsymbol{\gamma}_{i}^{T}{\bar{\mathbf{A}}^{i}}||_{2}+\lambda ||\boldsymbol{\gamma}_{i}||_{1}, s.t. \ {\boldsymbol{\gamma}_{i}}\geq 0   $$

The target of sparse representation is to find a sparse coefficient vector $\boldsymbol {\gamma }_{i} \in \mathbb {R}^{(N-1)}$, with $\mathbf {A}(i,\cdot)\approx \boldsymbol {\gamma }_{i}^{T}{\bar {\mathbf {A}}^{i}}$ and ||***γ***_*i*_||_1_ is minimized. ||***γ***_*i*_||_1_ is the *l*_1_ norm that sums the absolute values of ***γ***_*i*_, and minimizing ||***γ***_*i*_||_1_ can enforce ***γ***_*i*_ to be a sparse vector. *λ*(>0) is a scalar regularization parameter that balances the tradeoff between reconstruction error and sparsity of coefficients [34]. $\bar {\mathbf {A}}^{i} \in \mathbb {R}^{(N-1) \times |\mathcal {T}|}$ is a sub-matrix of **A** with the *i*-th row removed. In this way, **A**(*i*,·) is linearly reconstructed by other rows of **A**, instead of itself. ***γ***_*i*_(*j*) can be seen as the reconstruction contribution of **A**(*j*,·) to **A**(*i*,·). In other words, the larger the semantic similarity between **A**(*i*,·) and **A**(*j*,·), the larger the ***γ***_*i*_(*j*) is. Here, we solve the optimal ***γ***_*i*_ using the sparse learning with efficient projection package [39]. To further explain the usage of sparse representation to measure the semantic similarity between genes, we provide a simple workflow in Additional file 1: Figure S1.

Next, we employ ***γ***_*i*_ to define the semantic similarity between the *i*-th gene with respect to other genes, and use $\mathbf {S} \in \mathbb {R}^{N \times N}$ to store the semantic similarity between *N* genes. **S**(*i*,·) stores the similarity of the *i*-th gene with other genes, and it is defined as follows:

3 $$ \mathbf{S}(i,j)= \left\{ \begin{array}{l} \boldsymbol{\gamma}_{i}(j), \ \ \ \ \ \ \ \ \ \text{if}~j<i\\ \boldsymbol{\gamma}_{i}(j-1), \ \ \text{if}~j>i\\ 0, \quad \quad \quad \quad \text{otherwise} \\ \end{array} \right.   $$

By iteratively applying Eqs. (2–3) for *N* genes, we can sequentially fulfil each row of **S**. The similarity between a gene and itself is set as 0, since noisy annotations of a gene are predicted based on the annotations of semantic similar genes of that gene, instead of itself. To make **S** being a symmetric matrix, we set **S**=(**S** ^*T*^+**S**)/2. In fact, various approaches [34] utilize Eq. (3) to measure the similarity between samples, and find this similarity often performs better than many other widely-used similarity metrics, and is robust to noisy features.

A simple and intuitive idea to predict noisy annotations of a gene is to select neighborhood genes of a gene based on the semantic similarity between them and regard these genes as voters, and then to vote whether a term should be removed or not, based on the term’s association with these voters. The fewer votes the term obtains, the more likely the term as a noisy annotation of the gene is. In fact, this idea is widely used to aggregate annotations and to solve the disagreement between annotators [40, 41], and also adopted by NoisyGOA [32]. However, this idea does not differentiate varieties of neighborhood genes. To take into account these varieties, we use the semantic similarity derived from sparse representation to predict noisy annotations. If *t* is annotated to gene *i*, namely **A**(*i*,*t*)>0, the aggregated vote of *t* for the gene is counted as follows:

4 $$ \mathbf{V}_{SR}(i,t)={\sum}_{j=1}^{N} \mathbf{S}(i,j)\times \mathbf{A}(j,t)   $$

Equation (4) is similar to a weighted *k* nearest neighborhood (*k*NN) classifier [42], since **S**(*i*,·) is a sparse vector with most entries as (or close to) zeros and neighborhood genes of gene *i* are automatically determined by these nonzero entries. Equation (4) can be regarded as a weighted voting method and the weights are specified by the semantic similarity between them. If a term is annotated to a gene, but this term is not (or less frequently) annotated to that gene’s neighborhood genes than other terms, then this term has a larger probability as a noisy annotation of that gene than other terms. Here, we want to remark that if gene *i* has few similar genes, then all entries in **S**(*i*,·) will be equal or close to zeros. Consequently, terms annotated this gene are more likely to receive lower voting scores and to be identified as noisy annotations. Indeed, this extreme case is worthwhile for future pursue.

### Weighting annotations using evidence codes

Using aggregated votes to predict noisy annotations is a feasible solution [32, 41], but it does not take into account the differences among annotations. Evidence codes, attached with GO annotations, illustrate the sources where these annotations collected from. Some researchers used GO annotations archived on different periods to analyse the quality of annotations under different evidences codes [11, 21, 24], and found the quality varying among different branches and evidence codes. Motivated by these analysis, we estimate the ratios of noisy annotations for each evidence code in each branch and then employ the ratios to weight the gene-term association matrix **A**. Here, we collected two GOA files that archived on different months, then we take the annotations available in the former month but absent in the latter month as noisy annotations of the former GOA file. To account for GO change and its cascade influence on GO annotations, we only use the shared GO hierarchy in the two contemporary GO files. Let *N* ^*m*^(*c*) be the number of annotations attached with evidence code *c* in the *m*-th version GOA file, and $\bar {N}^{m}(c)$ be the number of noisy annotations tagged with evidence code *c* in that GOA file. The estimated ratio of noisy annotations for *c* can be approximated as:

5 $$ r_{ec}^{m}(c)= \frac{\bar{N}^{m}(c)}{N^{m}(c)}  $$

To more accurately estimate the ratio of noisy annotations for the *m*-th version, we sum up the ratios estimated from its *l* previous versions as follows:

6 $$ \tilde{r}^{m}_{ec}(c)=\frac{1}{l}\sum_{l'=m-l+1}^{m}r_{ec}^{l'}(c)  $$

Obviously, a large $\tilde {r}^{m}_{ec}(c)$ indicates annotations tagged with *c* are unstable and more likely to contain noisy annotations, since they change frequently in the previous versions. Based on $\tilde {r}^{m}_{ec}(c)$, we set different weights to different evidence codes as follows:

7 $$ w_{ec}(c)= \left\{ \begin{array}{l} 1, \ \text{if}~\tilde{r}^{m}_{ec}(c)<\tau\\ \theta, \ \text{otherwise} \\ \end{array} \right.   $$

*τ* is a threshold and set as the average value of $\tilde {r}^{m}_{ec}$ with respect to different evidence codes. Annotations tagged with evidence codes whose $\tilde {r}^{m}_{ec}(c)\geqslant \tau $ are unstable and likely to be noisy annotations. Therefore, we set *w*_*ec*_ of these annotations as *θ*(<1), and others as 1. Other specifications of *θ* and *τ* is postponed to be discussed in the next section.

GOC follow a convention to annotate genes with the appropriate and as well as specific terms that correctly describe the biology of the genes. The annotations stored in the GOA files are called *direct* annotations, and each of them is tagged with an evidence code. To make use of these direct annotations and evidence codes, if **A** ^*d*^(*i*,*t*) is tagged with evidence code *c*, we update the gene-term association matrix $\mathbf {A}^{d} \in \mathbb {R}^{N\times |\mathcal {T}|}$ as follows:

8 $$ \mathbf{A}^{d}_{ec}(i,t)= \mathbf{A}^{d}(i,t) \times w_{ec}(c)   $$

where **A** ^*d*^ is initialized by direct annotations only. If there are multiple evidence codes for the same gene-term association **A** ^*d*^(*i*,*t*), we set the maximal weight of these involved evidence codes to $\mathbf {A}^{d}_{ec}$.

Annotated with a term implies the gene also annotated with its ancestor terms via any path of GO hierarchy. In other words, if a gene is annotated with term *t*, this gene is inherently annotated with all the ancestors of *t*. This rule is called *true path rule* [1, 43]. To make use of this rule, we propagate the weights and extend $\mathbf {A}^{d}_{ec}$ to ancestor annotations of direct ones as follows:

9 $$ \mathbf{A}_{ec}(i,s)= max \left\{\mathbf{A}^{d}_{ec}(i,t) | s \in anc(t)\right\}   $$

where *a* *n* *c*(*t*) includes all ancestors of *t*. If ancestor annotation *s* is propagated from two or more direct annotations, we take maximal value of these direct annotations as the weight of **A**_*ec*_(*i*,*s*). This setting ensures the weights of ancestor annotations equal (or larger) than descendant annotations, since a descendant term describes more specific biological function than its ancestor terms and annotations with respect to ancestor terms are generally more easier to be verified than descendant ones. Another reason for this maximal setting is motivated by accumulated evidences from different sources. If the weight for an ancestor annotation is smaller than its descendant ones, the relevant term will be more likely to be identified as a noisy annotation than its descendants. This setting is not desirable. From the true path rule, if the ancestor term is not annotated to a gene, then all its descendants are not annotated to that gene, too.

### Noisy annotations prediction

To this end, we integrate the evidence weighted annotations in Eq. (9) and aggregated votes in Eq. (4) to predict noisy GO annotations of genes as follows:

10 $$ \mathbf{V}(i,t)=\alpha\times \mathbf{V}_{SR}(i,t)+(1-\alpha)\times \mathbf{A}_{ec}(i,t)   $$

where *α* is a scalar parameter to adjust the contribution of **V**_*SR*_ and **A**_*ec*_. If both *t* and *s* are annotated to the *i*-the gene and **V**(*i*,*t*)<**V**(*i*,*s*), then *t* is more likely to be a noisy annotation than *s*. Eq. (10) is motivated by the observation that if a term is annotated to a gene, but this term is not (or rarely) annotated to neighborhood genes of the gene and the evidence code attached with this annotation has a large estimated ratio of noisy annotations, then the annotation is more likely to be a noisy one. One shortcoming of Eq. (10) is that if a noisy annotation appears in successive GOA files and its relevant GO term is frequently annotated to neighborhood genes of the gene, this noisy annotation is difficult to be identified by NoGOA. This kind of noisy annotations are more challenging and remain for future pursue. To select a reasonable value for *α*, we can adjust it in the range [0, 1] by taking GOA files archived prior to the historical GOA files to train NoGOA and use the GOA files archived no late than the historical GOA files to validate the prediction. After that, we can select the optimal *α* to train NoGOA on the historical GOA files. Fortunately, our following empirical parameter sensitivity analysis shows that it is easy to select a reasonable and consistent *α* for NoGOA on GOA files of different species.

To predict noisy annotations, NoGOA not only takes advantage of sparse representation to reduce the interference of noisy annotations and of aggregated votes from neighborhood genes, but also weights annotations based on the estimated ratios of noisy annotations with respect to different evidence codes. Therefore, NoGOA has the potential to achieve better performance than using sparse representation or evidence codes alone. Our following experimental study corroborates this advantage and shows evidence codes can be used as a plugin with other semantic similarity based methods to improve the performance in predicting noisy annotations.

## Results and discussion

### Experimental protocols and comparing methods

We downloaded four versions of GOA files (archived in May and September) of six model species [44], *H. sapiens*, *A. thaliana*, *S. cerevisiae*, *G. gallus*, *B. Taurus* and *M. musculus* to comparatively study the performance of NoGOA and of other comparing methods in two successive years (2015 and 2016), respectively. To mitigate the impact of GO change in long intervals, we use the GO annotations archived in the first four months of the year (2015 or 2016) to estimate the ratio of noisy annotations for each evidence code and the annotations archived in May for prediction. We then validate the prediction based on annotations archived in September of the same year. Accordingly, we also downloaded contemporary GO files [45], which were archived on the same date as GOA files. To reduce the impact of evolved GO and annotations for evaluation, similar to the 2nd CAFA (Critical Assessment of protein Function Annotation algorithms) [5], we retain the terms that are included both in the historical and recent GO files, and filter out terms that are absent in historical or recent GO files. Next, these retained terms, direct annotations in the GOA files and the inherited ancestor annotations of these direct ones, are used to initialize the historical (archived in May) gene-term association matrix **A** ^*h*^ and recent (archived in September) gene-term matrix **A** ^*r*^, respectively. We consider the annotations available in **A** ^*h*^ but absent in **A** ^*r*^ as noisy annotations. To be honest, this consideration is not very good, because of the complicated evolutionary mechanism of GO and GO annotations [7, 11]. However, since noisy annotations are not readily available, we regard these removed annotations as ‘noisy annotations’ and use them to validate the predicted noisy annotations made by the comparing methods. The statistics of genes and annotations in 2015 and 2016 are listed in Tables 2 and 3. For instance, in 2016, there are 18,932 genes in H. sapiens and these genes are annotated with 13,172 BP GO terms. These genes in total have 1,141,456 annotations in BP branch, among them there are 22,706 noisy annotations.

**Table 2:** Statistics of GO annotations of *H. sapiens*, *A. thaliana*, *S. cerevisiae*, *G. gallus*, *B. Taurus* and *M. musculus* (archived date: May, 2015)

	Branch($|\mathcal {T}|$)	Annotations	Noisy annotations
H. sapiens(18939)	BP (13875)	1183415	23143
	CC (1672)	375982	2770
	MF (4244)	234599	2322
A. thaliana(24377)	BP (5132)	794092	2651
	CC (848)	222465	498
	MF (2684)	197422	2301
S. cerevisiae(5887)	BP (4768)	244374	898
	CC (931)	104831	87
	MF (2282)	65745	338
G. gallus(12782)	BP (11783)	572194	19603
	CC (1451)	201471	3859
	MF (3350)	144112	2345
B. Taurus(17316)	BP (11783)	768861	20788
	CC (1521)	272289	3745
	MF (3350)	189509	2371
M. musculus(21188)	BP (13744)	1036467	15376
	CC (1621)	356694	1603
	MF (4148)	231078	2195

The data in the parentheses of the 1st column is the number of genes, data in the 2nd column is the number of involved GO terms ($|\mathcal {T}|$), the 3rd column is the number of annotations for a particular branch, and the last column is the number of noisy annotations, which were available in the GOA file archived in May, but absent in the GOA file archived in September of the same year

**Table 3:** Statistics of GO annotations of *H. sapiens*, *A. thaliana*, *S. cerevisiae*, *G. gallus*, *B. Taurus* and *M. musculus* (archived date: May, 2016)

	branch($|\mathcal {T}|$)	Annotations	Noisy annotations
H. sapiens(18932)	BP (13172)	1141456	22706
	CC (1707)	385525	3141
	MF (4345)	243928	4660
A. thaliana(6931)	BP (4157)	243249	15918
	CC (750)	97616	2937
	MF (2271)	81318	3554
S. cerevisiae(6719)	BP (4385)	222754	13647
	CC (990)	108186	2768
	MF (2379)	65032	4394
G. gallus(10912)	BP (10643)	244374	898
	CC (1429)	177491	4448
	MF (3298)	124997	2130
B. Taurus(17886)	BP (11724)	753976	6541
	CC (1550)	281284	2244
	MF (3298)	194425	1396
M. musculus(21279)	BP (13141)	481417	18182
	CC (1686)	367461	3917
	MF (4238)	239664	2705

The data in the parentheses of the 1st column is the number of genes, data in the 2nd column is the number of involved terms ($|\mathcal {T}|$), the 3rd column is the number of annotations for a particular branch, and the last column is the number of noisy annotations, which were available in the GOA file archived in May, but absent in the GOA file archived in September of the same year

To comparatively study the performance of NoGOA, we take eight related methods as comparing methods. The details of these methods are introduced as follows:

- (i) *Random* randomly chooses a term annotated to a gene as the noisy annotation of that gene.
- (ii) *LF* randomly selects the term annotated to a gene but with the Lowest Frequency among *N* genes as the noisy annotation of the gene.
- (iii) *SR* is solely based on Sparse Representation [34] in Eq. (4) to predict noisy annotations.
- (iv) *EC* is solely based on Evidence Code to predict noisy annotations. More specifically, it chooses the term annotated to the *i*-th gene but with lowest weight in **A**_*ec*_(*i*,·) as a noisy annotation of the gene.
- (v) *NtN* is a semantic similarity based approach that can be adopted to predict noisy annotations [46]. It views each gene as a document and terms annotated to the gene as words of that document. It firstly utilizes the term-frequency, inverse document frequency in vector space model [47], and GO hierarchy to weight annotations located at different locations. Next, it employs singular value decomposition on the weighted gene-term association matrix and then chooses the term annotated to a gene but with lowest entry value in the decomposed matrix as a noisy annotation of that gene.
- (vi) *NoisyGOA* is originally proposed for predicting noisy annotations by our team [32]. It was elaborated in the last part of the 6th paragraph of Introduction section.
- (vii) *NtN+EC* integrates the predictions from evidence code updated gene-term association matrix **A**_*ec*_ (see Eq. (9)) and those from NtN (similar as Eq. (10)) to predict noisy annotations.
- (viii) *NoisyGOA+EC* integrates the predictions from **A**_*ec*_ and those from NoisyGOA (similar as Eq. (10)) to predict noisy annotations.

*λ*=0.5 is used in Eq. (2), and the parameters of NtN and NoisyGOA are fixed as the authors suggested in their original papers. In practice, we conducted experiments to study the sensitivity of *λ*∈[0.1,1] (as suggested by the package provider) [39] and found that NoGOA has stable performance in this range, so we use the median value *λ*=0.5 for experiment. In the following experiments, we denote the number of noisy annotations for gene *i* as *q*, and then take *q* entries with nonzero values in **A**(*i*,·) but with the smallest values in $\mathbf {V}(i,\cdot) \in \mathbb {R}^{|\mathcal {T}|}$ (see Eq. (10)) as the predicted noisy annotations of that gene. In this way, we can avoid genes having fewer neighborhood genes to receive systematically lower voting scores, since we determine noisy annotations by referring to **A**(*i*,·) and **V**(*i*,·), instead of all entries in **V**. To reach fair comparison, NoGOA and all other comparing methods use the same protocol to select *q* noisy annotations. This adopted protocol may affect the prediction of noisy annotations. Other more appropriate protocols are interesting future pursue. From the true path rule, if a term is not annotated to a gene, its descendant terms are also not annotated to this gene. To ensure consistency, if the descendant terms of the predicted *q* terms are annotated to the *i*-th gene, all the comparing methods will take descendant terms of these *q* terms as predicted noisy annotations of the gene, too.

To quantitatively analyze the performance of noisy annotations prediction, three metrics are adopted: *Precision*, *Recall* and *F1-Score*. The formal definitions of these metrics are provided as follows:

11 $$ p_{i}=\frac{TP_{i}}{TP_{i}+FP_{i}}, \ r_{i}=\frac{TP_{i}}{TP_{i}+FN_{i}}  $$

12 $$\text{Precision}=\frac{1}{N}\sum_{i=1}^{N} p_{i}, \ \text{Recall}=\frac{1}{N}\sum_{i=1}^{N} r_{i}  $$

13 $$ \text{F1-Score} =\frac{1}{N}\sum_{i=1}^{N} \frac{2\times p_{i} \times r_{i}}{p_{i} + r_{i}}  $$

where *T* *P*_*i*_ is the number of correctly predicted noisy annotations of the *i*-th gene, *F* *P*_*i*_ is the number of wrongly predicted noisy annotations, and *F* *N*_*i*_ is the number of noisy annotations not predicted by the predictor. *p*_*i*_ and *r*_*i*_ are the precision and recall on the *i*-th gene, they evaluate the fraction of predicted noisy annotations that are true noisy annotations and the fraction of noisy annotations that are correctly predicted, respectively. F1-Score firstly computes individual precision and recall for each gene, and then takes the average of harmonic mean of individual precision and recall of *N* genes.

### Results of predicting noisy annotations

In this section, we predict noisy annotations of genes based on the annotations in the historical GOA files, and then use the annotations in the recent GOA files to validate the predicted noisy annotations. Similar to CAFA2 [5], to get reliable and repeatable experimental results, we use bootstrapping to randomly take 85% genes and their annotations in the recent GOA files to validate the predicted noisy annotations. We independently repeat the above bootstrapping 500 times to avoid random effect. In these experiments, *α* in Eq. (10) is set as 0.2, and *θ* in Eq. (7) is set as 0.5. Other input values of *α* and *θ* will be discussed later. The recorded experiments results (average and standard deviation) on a particular species for a particular branch are revealed in Table 4 and Tables S1-S11 of the supplementary file. We use pairwise *t*-test at 95% significant level to check the difference among these comparing methods and highlight the best (or comparable best) performance in **boldface**.

**Table 4:** Performance of predicting noisy annotations in GOA files of *H. sapiens* (archived date: May, 2016)

		Random	LF	NtN	NoisyGOA	SR	EC	NtN+EC	NoisyGOA+EC	NoGOA
BP	Precision	23.99±0.49	29.50±0.57	23.71±0.47	33.98±0.67	35.24±0.56	29.43±0.56	26.30±0.51	38.55±0.72	**4** **1** **.** **1** **4**±0.76
	Recall	**5** **7** **.** **7** **5**±1.00	29.58±0.57	55.84±0.87	41.08±0.76	35.67±1.48	49.04±0.86	52.52±0.89	44.82±0.81	41.45±0.76
	F1-Score	31.51±0.60	29.54±0.57	30.94±0.55	36.63±0.70	35.44±0.69	35.04±0.64	33.24±0.61	**4** **0** **.** **9** **3**±0.75	**4** **1** **.** **2** **8**±0.76
CC	Precision	19.34±0.52	28.62±0.77	17.75±0.52	36.41±0.89	**4** **1** **.** **4** **1**±1.01	17.40±0.45	18.00±0.48	36.13±0.88	**4** **1** **.** **3** **4**±0.97
	Recall	50.62±1.12	28.69±0.77	49.68±1.18	44.45±1.02	41.91±1.02	**7** **9** **.** **2** **2**±1.40	44.80±1.07	44.15±1.02	41.85±0.98
	F1-Score	25.98±0.65	28.65±0.77	24.22±0.65	38.79±0.93	**4** **1** **.** **6** **3**±1.02	25.34±0.58	24.34±0.61	38.50±0.92	**4** **1** **.** **5** **6**±0.97
MF	Precision	27.74±0.39	23.60±0.38	36.43±0.45	38.16±0.48	46.18±0.54	41.25±0.50	49.90±0.55	52.18±0.57	**5** **8** **.** **9** **2**±0.60
	Recall	41.94±0.50	23.63±0.38	48.83±0.57	46.41±0.55	46.57±0.54	**6** **0** **.** **4** **6**±0.64	56.80±0.60	58.26±0.62	59.47±0.60
	F1-Score	30.35±0.41	23.61±0.38	38.82±0.47	39.44±0.48	46.34±0.54	44.45±0.51	51.75±0.56	53.23±0.58	**5** **9** **.** **1** **4**±0.60

The numbers in **boldface** denote the best performance

From these tables, we can easily observe that NoGOA achieves the best (or comparable best) performance among these comparing algorithms in most cases in terms of Precision and F1-score. NoisyGOA or NoisyGOA+EC get better performance than NoGOA on some species (such as *A. thaliana* in the BP branch (archived in May, 2015), and *G. gallus* in the BP branch (archived in May, 2016)), but NoGOA still obtains better results than other comparing approaches (Random, LF, NtN, EC and NtN+EC). This global observation validates the effectiveness of NoGOA in identifying noisy annotations. Both NoGOA and SR employ sparse representation to define the semantic similarity between genes and then use a *k*NN style algorithm to predict noisy annotations. SR often loses to NoGOA. This is principally because NoGOA additionally takes advantage of evidence codes to set different weights to different annotations. Similarly, NoGOA always gets better Precision and F1-score than EC, which predicts noisy annotations by only utilizing the evidence code weighted gene-term association matrix. This observation shows that integrating sparse representation with evidence code can generally improve the performance of noisy annotation prediction.

We adopt Wilcoxon signed rank test [48, 49] to assess the difference between NoGOA and these comparing algorithms with respect to F1-score on multiple species across three GO branches, and observe that NoGOA significantly works better than them with all the *p*-value smaller than 0.001. From these results, we can draw a conclusion that it is necessary and effective to integrate evidence codes with sparse representation for identifying noisy annotations. However, the F1-Score is between 34% and 74%, which means only a portion of noisy annotations can be correctly predicted and there is much space for future pursue.

Another observation from these tables is that EC has larger Recall than SR and NoGOA in most cases. The reason is that EC picks up terms with the lowest values in **A**_*ec*_(*i*,·) as noisy annotations, without considering the terms’ association with other genes. EC also takes descendant terms of these picked up terms as noisy annotations of the *i*-th gene and results in a large number of predicted noisy annotations. For this reason, it gets larger Recall but lower Precision than NoGOA, and loses to NoGOA on F1-score.

NtN also weights the gene-term association matrix by employing the GO hierarchy, but it does not consider the evidence codes attached with annotations. It frequently has large Recall but low Precision and F1-score. That is because NtN sets larger weights to specific terms (or annotations) than general ones, and the terms corresponding to general annotations are ranking ahead of specific ones as candidate noisy annotations. Because of true path rule, all the annotations with respect to descendant terms of these general terms are also deemed as noisy annotations by NtN. For this reason, NtN often gets larger Recall but much lower Precision and F1-score than other comparing methods.

Similar as SR, NtN and NoGOA, NoisyGOA also utilizes the semantic similarity between genes and it additionally uses taxonomic similarity between GO terms. NoisyGOA outperforms NtN, Random, and LF in many cases. This fact indicates taxonomic similarity is helpful for predicting noisy annotations. However, NoisyGOA is frequently outperformed by SR. This observation suggests that semantic similarity contributes much more than taxonomic similarity in predicting noisy annotations. NoisyGOA often loses to NoGOA. The reason is threefold: (i) NoGOA differentially treats neighborhood genes to aggregate votes, whereas NoisyGOA equally treats neighborhood genes; (ii) NoGOA takes advantage of evidence codes of annotations, while NoisyGOA does not; (iii) NoGOA adopts sparse representation to measure the semantic similarity between genes, which is less suffered from noisy annotations than the Cosine similarity adopted by NoisyGOA.

LF selects terms annotated to a gene but with the lowest frequency among *N* genes as noisy annotations of the gene. It frequently gets larger Precision and F1-score than Random and NtN. This observation indicates that the frequency of terms can be used as an important feature for predicting noisy annotations. In fact, NoGOA, SR and NoisyGOA also take advantage of this feature. More specifically, to determine whether a term should be annotated to a gene or not, they count how many times the term annotated to neighborhood genes of the gene.

Random randomly selects terms from all the terms annotated to a gene, and took these selected terms and their descendant terms as noisy annotations of that gene. It sometimes can get the largest Recall. That is principally because these randomly selected terms often have many descendants, which are also annotated to the same gene. Given the superior results of NoGOA to Random, LF and EC, we can conclude that noisy annotations are predictable.

To further study the rationality of using evidence codes, we also report the results of NoisyGOA+EC and NtN+EC in Table 1 and Additional file 1: Tables S1–S11. With the help of evidence codes, NoisyGOA+EC has improved performance than NoisyGOA, and NtN+EC also shows this pattern. These results show evidence codes can be used as a plugin to improve the performance of noisy annotation prediction. NoGOA performs significantly better than NoisyGOA+EC and NtN+EC. The fact again justifies the rationality of synergy SR with EC for predicting noisy annotations.

### Parameter sensitivity analysis

**Fig. 1:**
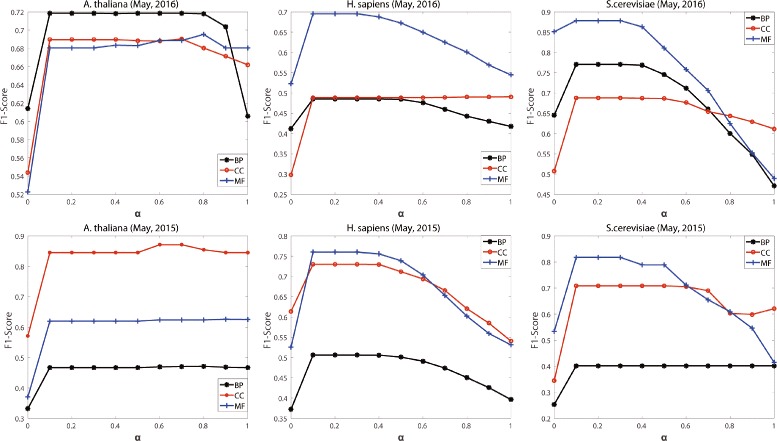
Performance of NoGOA in predicting noisy annotations under different input values of *α*

NoGOA are involved with three parameters *α* (in Eq. (10)), *τ* and *θ* (in Eq. (4)). We conduct additional experiments on GOA files of *H. sapiens*, *A. thaliana* and *S. cerevisiae* to study the sensitivity of NoGOA to these parameters and report the results in Fig. 1 (for *α*), Additional file 1: Figure S2 (for *θ*) and Additional file 1: Tables S12–S17 (for *τ*). When *α*=0, NoGOA is equivalent to EC. Likewise, when *α*=1, NoGOA is equivalent to SR.

In Fig. 1, we set *θ* as 0.5 and *τ* as the average of $r_{ec}^{m}$. There are 18 broken lines, and each of them denotes the change of F1-Scores under different input values of *α*. With the increase of *α*, these lines rise at first and then decrease (14 of 18) or keep stable. NoGOA always gets better results than the special case *α*=0 (or EC), and it also performs better than the special case *α*=1 (or SR). When *α*∈[0.1,0.3], NoGOA generally achieves better (or similar) performance than EC and SR across GOA files of different species archived in different years, so we set *α* as 0.2 for experiments. The sensitivity analysis of *α* further corroborates the necessity and advantage of integrating sparse representation with evidence codes. In some branches, F1-Scores remains relatively stable when *α*∈[0.1,1]. That is because SR plays a major role in noisy annotation prediction in these branches.

### Removing noisy annotations improves gene function prediction

To further study the influence of removing noisy annotations, we downloaded protein-protein interactions (PPI) network of *H. sapiens*, *A. thaliana* and *S. cerevisiae* from BioGrid [50] (archived date: 2016-05-01) for experiments. We take annotations whose aggregated scores **V**(*i*,*t*) smaller than 0.45 as predicted noisy annotations, and then update the gene-term association matrix **A**. From Eq. (10), for *α*=0.2 and *θ*=0.5, *α*×**V**_*SR*_(*i*,*t*)∈[0,0.2] and (1−*α*)×**A**_*ec*_(*i*,*t*)∈[0.4,0.8]. So we take the annotations with the lowest **A**_*ec*_(*i*,·) and **V**_*SR*_(*i*,·)<0.25 as noisy annotations of the *i*-th gene. Next, we apply a majority vote based function prediction model [51], which predicts GO annotations of a gene using the annotations of its interacting partners based on updated **A**. After that, we use the annotations in the recent GOA files to validate the predicted annotations. For comparison, we also apply the majority vote model on the same PPI network and the original **A**, and then follow the same protocol to evaluate the predictions. We label the latter method as ‘Original’.

To reach a comprehensive evaluation of gene function prediction, we use six evaluation metrics, namely *MicroAvgF1*, *MacroAvgF1*, *AvgPrec*, *AvgROC*, *Fmax* and *Smin*. These metrics have been applied to evaluate the results of gene function prediction [5, 36]. Except *Smin*, the higher the value of these metrics is, the better the performance is. These metrics measure the performance from different aspects, it is difficult for a method consistently better than others across all the metrics. The formal definitions of these metrics are provided in the supplementary file. The results with respect to *H. sapiens*, *A.thaliana* and *S. cerevisiae* are included in Table 5 and Additional file 1: Tables S18-S19.

**Table 5:** Results of gene function prediction on *H. sapiens* (archived date: May, 2016)

	BP		CC		MF	
	Original	NoGOA	Original	NoGOA	Original	NoGOA
MicroAvgF1	**92.85**	92.64	93.72	**93.92**	**93.10**	**93.10**
MacroAvgF1	89.04	**90.05**	88.06	**89.96**	89.55	**90.30**
AvgPrec	88.45	**88.50**	88.75	**89.19**	90.78	**90.81**
AvgROC	94.94	**96.73**	95.12	**96.66**	97.66	**98.35**
Fmax	**93.85**	93.50	93.85	**93.89**	94.62	**94.57**
Smin *↓*	8.69	**7.96**	**2.09**	**2.09**	2.40	**2.32**

The data in **boldface** denote the better result. ‘Original’ directly uses annotations in the historical GOA file to predict gene function; ‘NoGOA’ removes predicted noisy annotations from the historical GOA file and then predicts gene function. *↓* means the lower the value, the better the performance is

From the results in Table 5 and Additional file 1: Tables S18-S19, we can see that NoGOA has improved performance in gene function prediction than Original in most cases. We use Wilcoxon signed rank test to check the difference between the results of NoGOA and Original on these three model species, and find the *p*-value is smaller than 0.003.

From these results, we can draw a conclusion that removing noisy annotations improves the performance of gene function prediction.

### Real examples

To further investigate the ability of NoGOA in predicting noisy annotations of genes, we firstly study the number of predicted noisy annotations of *H. sapiens*, *A. thaliana* and *S. cerevisiae* for each evidence code. Since only direct annotations can obtain the sources and evidences in archived GOA files, we only count the numbers of direct noisy annotations, predicted noisy annotations and correctly predicted direct ones by NoGOA. These numbers are shown in Table S20-S25 of the supplementary file. Then, we take the first 4 genes (‘AAC1’,‘AAC3’,‘AAD14’,‘AAP1’), which have removed annotations in the recently archived (date: September 2016) GOA file of *S. cerevisiae* for illustrative study, and list the correctly (wrongly) predicted direct noisy annotations by NoGOA. The results of *S. cerevisiae* in CC branch are listed in Table 6. Other experimental results of *S. cerevisiae* in other branches are revealed in Additional file 1: Tables S26-S27.

**Table 6:** Examples of correctly (*√*) and wrongly(×) predicted direct noisy annotations by NoGOA in CC branch of *S. cerevisiae*

Protein		GO term	Evidence codes	Details
AAC1(ADP/ATP carrier)	*√*	GO:0005758 (mitochondrial intermembrane space)	TAS	Reactome:R-SCE-1252255
		GO:0005829 (cytosol)	TAS	Reactome:R-SCE-1252255
AAP1 (Alanine/arginine aminopeptidase)	*√*	GO:0005886 (plasma membrane)	IBA	GO_REF:0000033
		GO:0005664 (nuclear origin of replication recognition complex)	IDA	PMID:9372948
	×	GO:0000276 (mitochondrial proton-transporting ATP synthase complex, coupling factor F(o))	IDA	PMID:9224714

From Additional file 1: Tables S20–S25, we can find that the distribution of predicted noisy annotations for different evidence codes is often approximately consistent with the distribution of noisy annotations. This fact shows the effectiveness of NoGOA in identifying noisy annotations. The number of predicted noisy annotations is often larger than that of direct noisy annotations. That is because if an annotation is predicted as a noisy one of a gene, then its descendant annotations (if any) are also deemed as noisy annotations of that gene. Since the annotations expanded from GO hierarchy and direct annotations maybe supported by different evidence codes, we just report the correctly predicted direct noisy annotations here. In practice, by expanding these direct noisy annotations via the true path rule of GO, the number of correctly predicted noisy annotations can be sharply increased.

In most cases, IEA generally has much more noisy annotations than other evidence codes. That is mainly because the number of IEA annotations is the largest, and it does not mean that IEA annotations are the most unreliable. Similar to IEA, IBA also has many noisy annotations. TAS, IMP or IGI have more noisy annotations in BP than in MF and CC branches. EXP, ISA, ISO, ISM, RCA, IGC, IBD, IKR, IRD and IC annotations are relatively stable and have much fewer noisy annotations. The possible reason is that the number of annotations attached with these evidence codes is smaller than that of other evidence codes. These statistic numbers show that most evidence codes have no clear pattern of noisy annotations across all the GO branches. These numbers also support our motivation to adaptively set weights to annotations based on the estimated ratio of noisy annotations per evidence code, instead of presetting weights solely based on the categorization (i.e., Experimental and Computational) of evidence codes.

The selected 4 proteins have 16 direct noisy annotations in three branches. NoGOA predicts 20 noisy annotations, and 13 of them are correct. In actual fact, we rechecked the subsequent GOA files (till to February, 2017) of S. cerevisiae, and also found these 13 correctly predicted noisy annotations were always removed in these GOA files. It is anticipated that these correctly predicted noisy annotations could be confirmed by biological experiments. From Table 6 and Additional file 1: Tables S26-S27, we can find that these noisy annotations are attached with different evidence codes (IBA, IPI, IDA, IMP and TAS). In fact, these annotations are reviewed by curators, but they are not always more reliable than IEA [6, 8]. Another interesting observation is that, NoGOA only makes incorrect predictions on ‘AAP1’. The reason may be that compared with other genes, ‘AAP1’ contains more noisy annotations, which heavily mislead the semantic similarity between ‘AAP1’ and other genes.

## Conclusion

Current efforts toward computational gene function prediction are more focused on predicting GO annotations of un-annotated genes or replenishing missing annotations of partially annotated genes. Given the increasing application of GO annotations in various domains and misleading effect of noisy annotations, it is necessary to identify noisy annotations, which is a rarely studied but important open problem.

In this paper, we investigated whether noisy annotations are predictable or not, and how to predict noisy annotations. For this purpose, we introduced a method called NoGOA. NoGOA takes advantage of evidence codes attached with annotations and sparse representation to predict noisy annotations. Experimental results on six model species (H. sapiens, A. thaliana, S. cerevisiae, G. gallus, B. Taurus and M. musculus) show that noisy annotations are predictable and NoGOA can more accurately predict noisy annotations than other comparing algorithms. We believe our work will prompt more research toward removing noisy GO annotations.

## Additional file

Additional file 1 Supplementary file of ‘NoGOA: predicting noisy GO annotations using evidences and sparse representation’ This PDF file includes additional experimental results mentioned in the main text. (PDF 1300 kb)

## Abbreviations

BP: Biological process
CAFA: Critical assessment of protein function annotation
CC: Cellular component
EXP: Inferred from experiment
GO: Gene ontology
GOA: Gene ontology annotations
GOC: Gene ontology consortium
IBA: Inferred from biological aspect of ancestor
IBD: Inferred from biological aspect of descendant
IC: Inferred by curator
IDA: Inferred from direct assay
IEA: Inferred from electronic annotation
IEP: Inferred from expression pattern
IGC: Inferred from genomic context
IGI: Inferred from genetic interaction
IKR: Inferred from key residues
IMP: Inferred from mutant phenotype
IPI: Inferred from physical interaction
IRD: Inferred from rapid divergence
ISA: Inferred from sequence alignment
ISM: Inferred from sequence model
ISO: Inferred from sequence orthology
ISS: Inferred from sequence or structural similarity
MF: Molecular function
NAS: Non-traceable author statement
ND: No biological data available
PPI: Protein-Protein interactions
RCA: Inferred from reviewed computational analysis
TAS: Traceable author statement
