# Supplementary material for: NoGOA: predicting noisy GO annotations using evidences and sparse representation
Source: BMC Bioinformatics. 2017 Jul 21;18:350. doi: 10.1186/s12859-017-1764-z (PMC5521088; doi:10.1186/s12859-017-1764-z)
Supplement: Additional file 1 — Supplementary file of ‘NoGOA: predicting noisy GO annotations using evidences and sparse representation’ This PDF file includes additional experimental results mentioned in the main text. (PDF 1300 kb) [file 12859_2017_1764_MOESM1_ESM.pdf]

Supplementary file of 'NoGOA: predicting noisy GO annotations using evidences and sparse representation'

Guoxian Yu[1,*] Chang Lu[1], Jun Wang[1]
[1]College of Computer and Information Science, Southwest University, Chongqing 400715, China.
*Contact: gxyu@swu.edu.cn

July 5, 2017

1 Workflow of sparse representation

Fig. S1 illustratively shows the usage of sparse representation to compute the semantic similarity between genes based on GO annotations of them. In Fig. S1, **A** is a gene-term association matrix which contains

A	term1	term2	term3	term4	term5	term6	term7	term8	term9	term10
gene1	1	1	0	0	0	0	0	1	0	0
gene2	1	0	1	0	0	0	0	0	1	0
gene3	0	1	1	0	0	0	0	1	0	0
gene4	0	0	0	0	0	0	1	0	1	0
gene5	1	1	0	0	0	0	0	0	0	1
gene6	0	0	0	0	0	1	0	0	1	0
gene7	1	0	0	0	0	0	1	1	1	0
gene8	0	0	1	1	1	0	0	1	0	0

$$\hat{\gamma_i} = \arg\min_{\gamma_i} ||A(i,\cdot) - \gamma_i^T \bar{A}^i||_2 + \lambda||\gamma_i||_1$$
$$s.t.\ \gamma_i \geq 0$$

	gene2	gene3	gene4	gene5	gene6	gene7	gene8
γ_1^T	0	0.3074	0	0.3327	0	0.2169	0

	gene1	gene3	gene4	gene5	gene6	gene7	gene8
γ_2^T	0	0.016	0	0.0584	0.1002	0.3226	0.0356

	gene1	gene2	gene4	gene5	gene6	gene7	gene8
γ_3^T	0.4147	0	0	0	0	0	0.271

	gene1	gene2	gene3	gene5	gene6	gene7	gene8
γ_4^T	0	0	0	0	0.0773	0.3552	0

	gene1	gene2	gene3	gene4	gene6	gene7	gene8
γ_5^T	0.4942	0.0058	0	0	0	0	0

	gene1	gene2	gene3	gene4	gene5	gene7	gene8
γ_6^T	0	0.1053	0	0.1974	0	0	0

	gene1	gene2	gene3	gene4	gene5	gene6	gene8
γ_7^T	0.4626	0.1142	0	0.6929	0	0	0

	gene1	gene2	gene3	gene4	gene5	gene6	gene7
γ_8^T	0	0.0055	0.4937	0	0	0	0.0043

$$S(i,j) = \begin{cases} \gamma_i(j), & \text{if } j < i \\ \gamma_i(j-1), & \text{if } j > i \\ 0, & \text{otherwise} \end{cases}$$

S	gene1	gene2	gene3	gene4	gene5	gene6	gene7	gene8
gene1	0	0	0.3074	0	0.3327	0	0.2169	0
gene2	0	0	0.016	0	0.0584	0.1002	0.3226	0.0356
gene3	0.4147	0	0	0	0	0	0	0.271
gene4	0	0	0	0	0	0.0773	0.3552	0
gene5	0.4942	0.0058	0	0	0	0	0	0
gene6	0	0.1053	0	0.1974	0	0	0	0
gene7	0.4626	0.1142	0	0.6929	0	0	0	0
gene8	0	0.0055	0.4937	0	0	0	0.0043	0

Figure S1: A workflow of using sparse representation to compute semantic similarity between genes

8 genes and 10 GO terms. By minimizing the first Equation in Fig. S1, we can obtain $\{\gamma_i\}_{i=1}^{8}$, which make $\gamma_i^T \bar{A}^i$ close to $\mathbf{A}(i,\cdot)$ and $||\gamma_i||_1$ is minimized. For example, γ_1 means that $\mathbf{A}(1,\cdot)$ is reconstructed by $0.3074 \times \mathbf{A}(3,\cdot)$, $0.3327 \times \mathbf{A}(5,\cdot)$ and $0.2069 \times \mathbf{A}(7,\cdot)$, so the similarity between $\mathbf{A}(1,\cdot)$ and $\{\mathbf{A}(i,\cdot)\}_{i=2}^{8}$ are 0, 0.3074, 0, 0.3327, 0, 0.2069 and 0, respectively. Since we will not use the similarity between a gene and itself, we set the diagonal entry of **S** as 0. Finally, we set $\mathbf{S} = (\mathbf{S}^T + \mathbf{S})/2$ to ensure **S** be a symmetric matrix and store the semantic similarity between genes in **S**.

2 Performance of noisy annotations prediction

In the main text, we reported the results of eight comparing methods, LF, SR, EC, NtN, NoisyGOA, NtN+EC, NoisyGOA+EC and NoGOA in predicting the noisy annotations of genes on achieved GOA files of *H. Sapiens* (archived date: May 2016). Here, the results on *A. thaliana* and *S. cerevisiae* and *H. Sapiens*, *G. gallus*, *B. Taurus* and *M. musculus*(archived date: May 2015 or May 2016) are included in Tables S1-S11. In these tables, the statistical best performer is highlighted in **boldface** and significance

is checked by pairwise t-test at 95% level. Obviously, these results provide the similar observations and conclusions as in the main text.

Table S1: Performance of predicting noisy annotations in *A. thaliana* on archived GOA file (archived date: May, 2016). The numbers in **boldface** denote the best performance.

		Random	LF	NtN	NoisyGOA	SR	EC	NtN+EC	NoisyGOA+EC	NoGOA
BP	Precision	35.67±0.54	43.69±0.63	34.70±0.53	47.18±0.74	51.02±0.74	46.34±0.67	40.59±0.63	54.68±0.80	**60.52±0.80**
	Recall	66.36±0.88	43.82±0.64	**69.74±0.90**	55.14±0.84	52.02±0.75	67.02±0.88	67.12±0.89	61.88±0.89	61.68±0.81
	F1-Score	43.26±0.62	43.74±0.64	42.54±0.60	50.03±0.77	51.44±0.74	52.17±0.71	47.71±0.70	57.31±0.83	**61.02±0.80**
CC	Precision	35.88±0.87	46.22±1.22	36.47±0.91	45.24±1.08	55.70±1.23	37.21±0.94	37.26±0.95	47.61±1.13	**58.10±1.32**
	Recall	67.89±1.50	46.29±1.22	69.16±1.53	65.86±1.48	57.14±1.26	**82.94±1.71**	65.07±1.49	66.87±1.51	59.44±1.34
	F1-Score	43.94±1.01	46.25±1.22	44.85±1.06	50.90±1.18	56.32±1.24	46.31±1.08	44.99±1.08	52.96±1.23	**58.67±1.33**
MF	Precision	47.28±1.05	55.05±1.28	48.89±1.09	52.82±1.28	**56.97±1.27**	44.09±1.06	48.81±1.11	52.69±1.22	**57.00±1.27**
	Recall	**74.18±1.50**	55.17±1.29	70.39±1.48	64.59±1.50	59.31±1.41	71.94±1.63	66.60±1.42	64.05±1.44	59.02±1.31
	F1-Score	55.44±1.18	55.10±1.29	55.77±1.21	56.18±1.36	**57.75±1.30**	52.00±1.21	54.86±1.21	56.90±1.29	**57.85±1.29**

Table S2: Performance of predicting noisy annotations in *S. cerevisiae* on archived GOA file (archived date: May, 2016). The numbers in **boldface** denote the best performance.

		Random	LF	NtN	NoisyGOA	SR	EC	NtN+EC	NoisyGOA+EC	NoGOA
BP	Precision	24.11±0.39	32.21±0.50	23.50±0.38	35.02±0.56	39.66±0.57	50.34±0.67	32.29±0.46	62.15±0.76	**65.15±0.76**
	Recall	58.99 ±0.73	32.32±0.50	58.49±0.74	42.12±0.65	40.60±0.58	**66.68±0.81**	62.32±0.77	**66.23±0.79**	65.94±0.76
	F1-Score	31.14±0.45	32.26±0.50	30.36±0.44	37.53±0.59	40.08±0.58	54.81±0.70	39.37±0.52	63.72±0.77	**65.50±0.76**
CC	Precision	25.47±0.61	38.12±0.90	26.99±0.67	37.87±0.93	51.28±1.12	37.33±0.89	31.99±0.78	45.97±1.05	**57.90±1.20**
	Recall	64.05±1.21	38.28±0.91	64.26±1.30	49.44±1.14	52.80±1.15	**72.71±1.40**	61.63±1.28	54.77±1.19	59.23±1.23
	F1-Score	33.16±0.72	38.19±0.91	34.81±0.79	41.38±0.98	51.95±1.13	43.10±0.96	39.06±0.89	48.73±1.09	**58.48±1.21**
MF	Precision	29.90±0.42	25.29±0.42	30.02±0.43	40.40±0.56	41.38±0.51	70.79±0.62	70.76±0.68	73.42±0.71	**74.60±0.69**
	Recall	46.46±0.55	25.31±0.42	46.90±0.56	41.88±0.57	41.90±0.52	**78.15±0.66**	76.05±0.71	74.34±0.71	74.80±0.69
	F1-Score	33.23±0.43	25.30±0.42	33.66±0.45	40.92±0.57	41.59±0.51	72.38±0.62	72.24±0.68	73.75±0.71	**74.69±0.69**

Table S3: Performance of predicting noisy annotations in *B. Taurus* on archived GOA file (archived date: May, 2016). The numbers in **boldface** denote the best performance.

		Random	LF	NtN	NoisyGOA	SR	EC	NtN+EC	NoisyGOA+EC	NoGOA
BP	Precision	27.69±0.84	32.53±1.00	28.33±0.84	22.80±0.46	39.59±1.22	28.87±0.91	29.21±0.87	35.98±1.07	**41.37±1.20**
	Recall	59.28±1.52	32.53±1.00	59.97±1.49	59.17±1.03	39.99±1.23	**60.40±1.62**	58.93±1.53	43.86±1.26	41.80±1.21
	F1-Score	34.83±0.98	32.53±1.00	35.16±0.98	30.46±0.57	39.77±1.22	36.19±1.06	35.90±1.01	38.87±1.14	**41.57±1.20**
CC	Precision	31.50±0.93	37.30±1.19	29.65±0.93	38.28±1.10	50.96±1.34	41.27±1.21	36.37±1.11	46.15±1.26	**55.90±1.47**
	Recall	59.88±1.54	37.40±1.19	57.64±1.54	51.19±1.39	52.35±1.39	**76.83±1.89**	56.46±1.61	56.66±1.47	57.26±1.50
	F1-Score	38.34±1.06	37.35±1.19	36.64±1.06	42.03±1.17	51.56±1.36	47.77±1.32	42.14±1.24	49.20±1.31	**56.49±1.48**
MF	Precision	33.69±1.07	40.00±1.33	32.19±1.07	45.23±1.32	50.33±1.45	32.71±1.05	34.88±1.12	47.99±1.36	**53.09±1.49**
	Recall	58.48±1.69	40.20 ±1.33	53.96±1.60	54.68±1.53	51.06±1.46	**69.51±1.74**	55.63±1.62	57.36±1.58	53.82±1.51
	F1-Score	40.10±1.22	40.09±1.33	38.06±1.19	48.02±1.36	50.66±1.46	40.26±1.18	40.74±1.25	50.75±1.41	**53.42±1.50**

Table S4: Performance of predicting noisy annotations in *G. gallus* on archived GOA file (archived date: May, 2016). The numbers in **boldface** denote the best performance.

		Random	LF	NtN	NoisyGOA	SR	EC	NtN+EC	NoisyGOA+EC	NoGOA
BP	Precision	43.74±1.02	50.03±1.20	43.82±1.04	40.91±0.74	53.75±1.29	52.60±1.26	48.29±1.07	60.55±1.37	**61.46±1.33**
	Recall	71.81±1.48	50.05±1.20	71.99±1.50	48.01±0.83	54.35±1.30	**72.45±1.60**	71.68±1.49	66.09±1.47	61.93±1.34
	F1-Score	51.37±1.14	50.04±1.20	51.57±1.16	43.45±0.77	54.02±1.30	58.47±1.35	55.31±1.18	**62.74±1.40**	61.68±1.34
CC	Precision	41.91±1.03	49.36±1.12	41.51±1.02	48.26±1.12	59.61±1.25	53.78±1.17	48.17±1.04	57.82±1.27	**65.29±1.37**
	Recall	68.50±1.45	49.44±1.12	68.22±1.51	61.78±1.37	61.29±1.28	**80.76±1.51**	69.94 ±1.37	67.52±1.44	66.63±1.39
	F1-Score	49.03±1.14	49.40±1.12	48.83±1.15	52.47±1.19	60.31±1.26	59.57±1.23	54.60±1.13	60.79±1.32	**65.85±1.38**
MF	Precision	38.57±1.10	49.06±1.37	39.18±1.14	50.75±1.38	56.11±1.40	38.06±1.08	41.44±1.17	53.24±1.40	**58.15±1.52**
	Recall	66.00±1.69	49.18±1.38	65.58±1.70	58.76±1.55	57.34±1.43	**73.44±1.67**	65.36±1.63	61.51±1.58	59.37±1.54
	F1-Score	45.63±1.24	49.11±1.37	46.38±1.27	53.21±1.43	56.65±1.41	45.69±1.20	48.13±1.29	55.80±1.45	**58.69±1.53**

Table S5: Performance of predicting noisy annotations in *M. musculus* on archived GOA file (archived date: May, 2016). The numbers in **boldface** denote the best performance.

		Random	LF	NtN	NoisyGOA	SR	EC	NtN+EC	NoisyGOA+EC	NoGOA
BP	Precision	23.07±0.49	26.43±0.57	22.80±0.46	34.21±0.65	32.29±0.70	40.45±0.75	28.37±0.60	46.66±0.92	**50.20±0.97**
	Recall	56.78±1.02	26.45±0.57	59.17±1.03	41.22±0.75	32.62±0.71	**60.94±1.01**	56.43±1.05	56.00±1.05	50.55±0.97
	F1-Score	30.50±0.60	26.44±0.57	30.46±0.57	36.73±0.68	32.44±0.70	45.75±0.81	35.85±0.72	49.07±0.95	**50.36±0.97**
CC	Precision	23.32±0.56	30.69±0.71	21.17±0.52	34.25±0.80	42.95±0.93	45.00±0.91	30.48±0.68	52.09±1.02	**57.61±1.11**
	Recall	56.61±1.13	30.83±0.72	57.37±1.15	43.21±0.95	43.73±0.95	**66.52±1.25**	56.28±1.10	57.47±1.09	57.95±1.12
	F1-Score	30.51±0.67	30.75±0.71	28.37±0.64	37.14±0.84	43.29±0.94	49.83±0.97	36.95±0.77	53.91±1.04	**57.76±1.11**
MF	Precision	28.27±0.64	29.98±0.69	28.77±0.68	43.84± 0.89	46.60±0.90	40.28±0.83	42.31±0.91	56.02±1.02	**56.86±1.02**
	Recall	50.85±0.99	29.98±0.69	52.04±1.05	47.99±0.95	47.02±0.90	58.44±1.08	54.66±1.08	**59.22±1.06**	57.34±1.03
	F1-Score	33.29±0.70	29.98±0.69	33.63±0.75	44.97±0.90	46.79±0.90	44.18±0.87	45.77±0.95	**57.00±1.03**	**57.06±1.02**

Table S6: Performance of predicting noisy annotations in *A. thaliana* on archived GOA file (archived date: May, 2015). The numbers in **boldface** denote the best performance.

		Random	LF	NtN	NoisyGOA	SR	EC	NtN+EC	NoisyGOA+EC	NoGOA
BP	Precision	27.19±0.74	36.59±0.95	19.81±0.38	**40.01±1.12**	39.85±1.12	23.25±0.72	22.93±0.81	**41.05±1.11**	39.39±1.09
	Recall	47.89±1.17	36.76±0.95	49.27±0.75	**57.48±1.41**	40.37±1.13	52.84±1.30	38.73±1.16	**58.21±1.41**	39.91±1.11
	F1-Score	31.48±0.81	36.66±0.95	26.06±0.45	**43.04±1.17**	40.08±1.12	28.20±0.79	26.40±0.88	**43.95±1.16**	39.62±1.10
CC	Precision	40.37±2.58	52.15±2.70	19.16±0.57	58.93±3.10	**72.78±3.66**	41.40±2.42	43.49±2.51	59.46±3.11	**71.51±3.58**
	Recall	67.44±3.82	52.15±2.70	52.27±1.28	77.76±3.71	72.48±3.69	**81.04±3.80**	65.01±3.37	76.07±3.69	72.20±3.60
	F1-Score	47.09±2.85	52.15±2.70	26.10±0.71	63.66±3.22	**72.07±3.67**	48.77±2.65	49.44±2.72	64.07±3.25	**71.80±3.59**
MF	Precision	26.13±0.53	51.80±0.94	24.02±0.58	32.59±0.65	**52.84±0.93**	22.06±0.42	22.86±0.43	32.28±0.65	**52.53±0.96**
	Recall	57.08±1.01	51.80±0.94	47.63±1.01	61.61±1.04	53.08±0.93	**73.12±1.12**	65.12±1.04	61.30±1.04	52.67±0.96
	F1-Score	33.66±0.63	51.80±0.94	29.65±0.67	38.68±0.72	**52.80±0.93**	31.43±0.54	31.59±0.55	38.38±0.71	**52.59±0.96**

Table S7: Performance of predicting noisy annotations in *H. sapiens* on archived GOA file (archived date: May, 2015). The numbers in **boldface** denote the best performance.

		Random	LF	NtN	NoisyGOA	SR	EC	NtN+EC	NoisyGOA+EC	NoGOA
BP	Precision	22.81±0.35	30.03±0.45	19.81±0.38	31.55±0.55	33.45±0.56	26.61±0.47	22.34±0.41	38.45±0.65	**42.74±0.64**
	Recall	**56.55±0.72**	30.09±0.45	49.27±0.75	37.83±0.64	34.03±0.57	44.58±0.68	48.32±0.74	44.38±0.72	43.45±0.65
	F1-Score	29.92±0.43	30.06±0.45	26.06±0.45	33.80±0.58	33.70±0.56	31.62±0.53	28.58±0.49	40.58±0.67	**43.04±0.64**
CC	Precision	19.14±0.54	33.97±0.83	19.16±0.57	41.12±1.00	45.70±1.07	48.25±1.10	35.94±0.90	59.28±1.26	**61.77±1.34**
	Recall	51.07±1.19	34.00±0.83	52.27±1.28	50.46±1.16	46.39±1.09	62.61±1.35	57.75±1.31	**64.76±1.36**	62.23±1.35
	F1-Score	25.84±0.67	33.98±0.83	26.10±0.71	43.93±1.04	46.00±1.08	52.18±1.16	42.02±1.00	61.12±1.29	**61.98±1.35**
MF	Precision	22.23±0.55	35.53±0.80	24.02±0.58	41.12±0.92	44.99±0.95	40.75±0.85	41.31±0.84	60.81±1.12	**64.46±1.22**
	Recall	49.58±1.08	35.74±0.81	47.63±1.01	43.24±0.96	45.36±0.96	56.92±1.09	55.21±1.01	62.47±1.14	**64.77±1.22**
	F1-Score	28.45±0.66	35.61±0.80	29.65±0.67	41.83±0.93	45.15±0.95	44.61±0.90	45.37±0.88	61.38±1.13	**64.59±1.22**

Table S8: Performance of predicting noisy annotations in *S. cerevisiae* on archived GOA file (archived date: May, 2015). The numbers in **boldface** denote the best performance.

		Random	LF	NtN	NoisyGOA	SR	EC	NtN+EC	NoisyGOA+EC	NoGOA
BP	Precision	20.54±1.07	21.85±1.19	16.75±1.03	30.64±1.67	**34.00±1.69**	16.73±1.02	19.43±1.14	30.70±1.50	**34.14±1.68**
	Recall	**49.90±1.95**	21.85±1.19	38.83±1.82	35.45±1.85	34.21±1.70	44.88±2.10	45.46±1.94	35.51±1.68	34.34±1.69
	F1-Score	25.64±1.21	21.85±1.19	20.90±1.16	32.18±1.72	**34.10±1.69**	21.51±1.17	23.69±1.25	32.24±1.55	**34.23±1.69**
CC	Precision	16.65±2.24	38.49±4.22	16.94±2.31	33.34±4.34	52.61±5.47	23.72±3.44	19.93±2.93	41.86±5.22	**59.77±6.28**
	Recall	58.41±6.30	38.59±4.22	61.91±6.35	41.76±5.33	52.99±5.51	**74.21±7.19**	48.83±5.57	49.81±6.07	59.98±6.30
	F1-Score	23.71±2.96	38.54±4.22	23.98±3.00	35.94±4.61	52.79±5.49	29.25±3.82	25.52±3.43	44.33±5.46	**59.87±6.29**
MF	Precision	23.08±1.47	30.72±1.95	22.85±1.51	22.85±0.02	35.11±2.17	43.49±2.45	38.02±2.21	64.43±3.06	**69.22±3.24**
	Recall	47.89±2.52	30.72±1.95	44.85±2.60	44.85±0.03	35.63±2.20	52.51±2.73	46.57±2.57	65.14±3.07	**69.82±3.25**
	F1-Score	28.79±1.70	30.72±1.95	28.27±1.76	28.27±0.02	35.29±2.18	45.66±2.50	40.39±2.28	64.66±3.06	**69.44±3.24**

Table S9: Performance of predicting noisy annotations in *B. Taurus* on archived GOA file (archived date: May, 2015). The numbers in **boldface** denote the best performance.

		Random	LF	NtN	NoisyGOA	SR	EC	NtN+EC	NoisyGOA+EC	NoGOA
BP	Precision	27.40±0.51	32.83±0.58	28.33±0.84	37.04±0.66	38.45±0.65	29.95±0.52	28.62±0.50	37.63±0.64	**39.94±0.68**
	Recall	55.43±0.88	32.88±0.58	**59.97±1.50**	43.90±0.74	38.98±0.66	56.64±0.87	55.07±0.80	44.93±0.73	40.49±0.69
	F1-Score	33.95±0.59	32.85±0.58	35.17±0.98	39.54±0.68	38.68±0.65	36.47±0.59	35.11±0.57	**40.24±0.67**	**40.18±0.68**
CC	Precision	36.70±0.90	48.52±1.10	37.87±0.92	49.12±1.09	55.80±1.15	35.44±0.84	37.59±0.87	50.55±1.12	**56.91±1.21**
	Recall	65.01±1.38	48.83±1.11	66.24±1.35	63.59±1.39	57.33±1.18	**78.20±1.50**	62.89±1.31	64.98±1.41	58.46±1.24
	F1-Score	43.26±1.00	48.65±1.10	44.59±1.01	53.27±1.16	56.44±1.16	43.22±0.95	44.09±0.97	54.66±1.18	**57.56±1.22**
MF	Precision	30.62±0.69	41.24±0.94	32.38±0.86	49.36±1.04	52.40±1.05	28.38±0.67	32.97±0.74	48.95±1.02	**53.93±1.05**
	Recall	58.76±1.14	41.38±0.94	63.90±1.46	53.83±1.09	52.96±1.06	**66.59±1.34**	56.95±1.13	53.63±1.09	54.33±1.06
	F1-Score	37.18±0.78	41.29±0.94	39.37±0.97	50.79±1.06	52.64±1.05	35.77±0.78	39.38±0.83	50.44±1.04	**54.11±1.06**

Table S10: Performance of predicting noisy annotations in *G. gallus* on archived GOA file (archived date: May, 2015). The numbers in **boldface** denote the best performance.

		Random	LF	NtN	NoisyGOA	SR	EC	NtN+EC	NoisyGOA+EC	NoGOA
BP	Precision	30.44±0.60	35.79±0.66	30.85±0.57	40.19±0.74	42.31±0.74	32.17±0.58	31.75±0.60	41.29±0.74	**43.49±0.76**
	Recall	59.16±0.97	35.79±0.66	57.81±0.93	48.01±0.83	42.79±0.75	**58.80±0.94**	55.81±0.90	48.01±0.86	43.95±0.77
	F1-Score	37.22±0.68	35.79±0.66	37.30±0.65	43.45±0.77	42.53±0.74	38.73±0.66	37.96±0.67	**43.86±0.78**	**43.70±0.76**
CC	Precision	40.52±0.91	50.84±1.19	44.34±1.04	53.41±1.19	57.30±1.19	39.66±0.94	45.51±1.05	53.78±1.20	**58.16±1.23**
	Recall	66.39±1.32	50.87±1.19	65.34±1.40	67.32±1.42	58.79±1.21	**80.30±1.56**	65.13±1.40	67.57±1.42	59.60±1.25
	F1-Score	46.97±1.00	50.85±1.19	50.03±1.12	57.62±1.25	57.91±1.20	46.86±1.04	51.09±1.13	57.94±1.26	**58.75±1.24**
MF	Precision	33.24±0.83	44.80±1.07	31.28±0.75	52.00±1.17	53.84±1.20	30.58±0.80	33.09±0.81	50.92±1.07	**55.46±1.23**
	Recall	62.07±1.29	44.94±1.07	56.56±1.18	55.68±1.23	54.57±1.21	**68.12±1.44**	62.18±1.26	55.04±1.44	56.11±1.24
	F1-Score	39.95±0.93	44.85±1.07	37.82±0.85	53.25±1.19	54.15±1.21	38.00±0.91	39.82±0.90	52.29±1.09	**55.74±1.23**

Table S11: Performance of predicting noisy annotations in *M. musculus* on archived GOA file (archived date: May, 2015). The numbers in **boldface** denote the best performance.

		Random	LF	NtN	NoisyGOA	SR	EC	NtN+EC	NoisyGOA+EC	NoGOA
BP	Precision	21.50±0.44	26.89±0.59	22.89±0.52	34.21±0.65	34.45±0.69	46.21±0.83	27.06±0.52	57.26±0.89	**59.50±0.96**
	Recall	48.87±0.81	26.90±0.59	50.48±0.92	41.22±0.75	34.82±0.69	60.79±1.02	51.99±0.86	**62.33±0.94**	59.99±0.97
	F1-Score	27.41±0.51	26.89±0.59	28.87±0.60	36.73±0.68	34.62±0.69	50.72±0.89	33.27±0.59	59.19±0.91	**59.71±0.97**
CC	Precision	26.77±0.80	37.85±1.02	26.91±0.82	44.94±1.17	46.61±1.23	52.63±1.29	41.25±1.08	64.59±1.52	**65.26±1.49**
	Recall	57.29±1.44	37.92±1.02	59.66±1.56	56.77±1.41	47.45±1.25	68.19±1.55	61.22±1.46	**71.75±1.64**	66.17±1.51
	F1-Score	33.55±0.92	37.88±1.02	34.11±0.97	48.64±1.23	46.96±1.24	57.03±1.35	47.42±1.18	**67.06±1.55**	65.65±1.49
MF	Precision	23.09±0.60	35.14±0.87	25.31±0.60	43.89±1.02	45.16±1.02	42.84±0.97	42.59±0.91	63.43±1.25	**67.89±1.29**
	Recall	48.75±1.09	35.22±0.87	56.42±1.11	47.77±1.08	45.42±1.03	62.09±1.23	61.60±1.19	66.13±1.29	**68.16±1.29**
	F1-Score	29.04±0.71	35.18±0.87	32.22±0.71	44.95±1.04	45.28±1.03	47.19±1.01	47.55±0.97	64.22±1.26	**68.00±1.29**

3 Parameter sensitivity analysis

In order to study the effectiveness of NoGOA under different input values of θ, we vary θ from 0.1 to 0.9 while fixing α as 0.2. The results are shown in Fig. S2. From the results in Fig. S2, we can find that F1-Score remains stable when $\theta \in [0.1, 0.7]$ and then decreases when $\theta \in [0.7, 0.9]$ in most cases. These results suggest that the weight for evidence codes whose estimated ratios of noisy annotations above the threshold τ, and for those whose estimated ratios below τ should be differently specified. The sensitivity study of θ again justifies the rationality of setting different weights to different annotations based on the estimated ratio of noisy annotations per evidence code.

We also study the effectiveness of NoGOA under different specifications of τ, we report the results of NoGOA in predicting noisy annotations when τ is the average (or median) of r_{ec}^m. In fact, we also test $\tau = 0$ for NoGOA, since annotations with respect to more than half evidence codes in the historical GOA files do not disappear in the recent GOA files, the results of NoGOA with $\tau = 0$ are the same as NoGOA with τ equal to the median of r_{ec}^m. The results are shown in Tables S12-S17.

From these tables, we can find that τ influences the performance of predicting noisy annotations. In most cases, when $\tau = 0$, NoGOA gets lower performance. That is because the evidence codes which do not have noisy annotations always have few annotations. NoGOA assigns larger weights to annotations tagged with these evidence codes, and smaller weight to others. As a result, using 0 to differentiate noisy

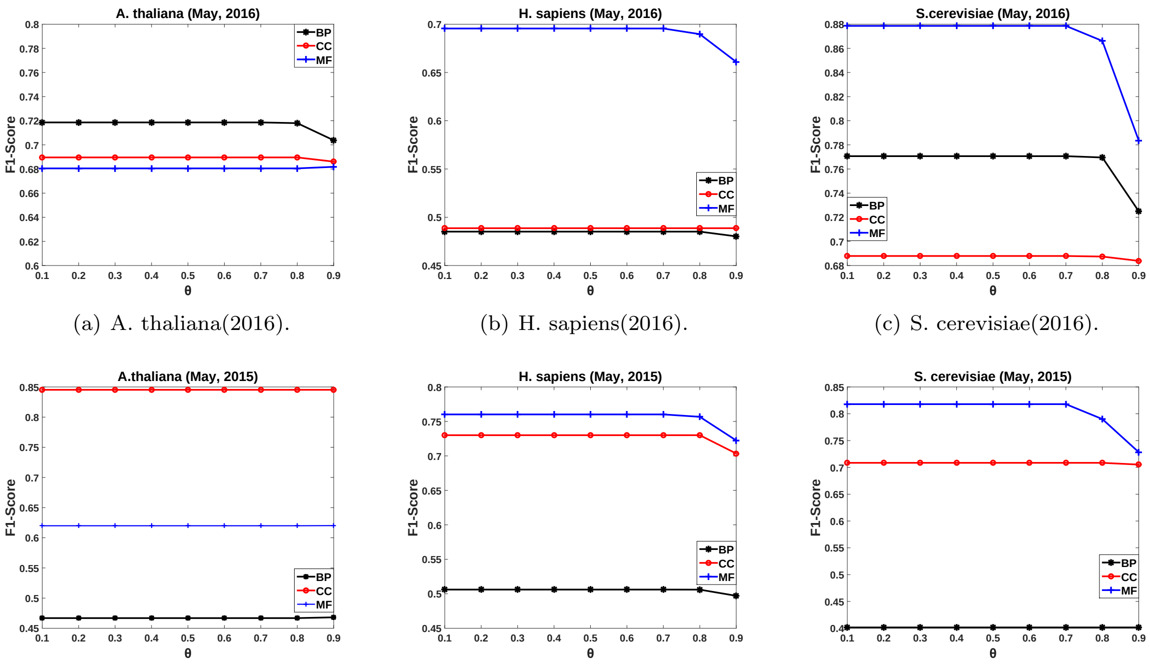

(a) A. thaliana(2016). (b) H. sapiens(2016). (c) S. cerevisiae(2016).

(d) A. thaliana(2015). (e) H. sapiens(2015). (f) S. cerevisiae(2015).

Figure S2: Performance of NoGOA in predicting noisy annotations under different input values of θ.

annotations from true annotations makes the performance un-obvious.

Table S12: Performance of NoGOA in predicting noisy annotation under different values of τ in *A. thaliana* on archived GOA files (May, 2016). The numbers in **boldface** denote the better performance.

		Median(0)	Average
BP	Precision	51.04±0.76	**60.52±0.80**
	Recall	52.04±0.77	**61.68±0.81**
	F1-Score	51.46±0.76	**61.02±0.80**
CC	Precision	55.85±1.28	**58.10±1.32**
	Recall	57.29±1.32	**59.44±1.34**
	F1-Score	56.47±1.30	**58.67±1.33**
MF	Precision	**61.29±1.33**	57.00±1.27
	Recall	**63.34±1.37**	59.02±1.31
	F1-Score	**62.15±1.34**	57.85±1.29

Table S13: Performance of predicting noisy annotations on different τ in *H. sapiens* on archived GOA files (May, 2016). The numbers in **boldface** denote the best performance.

		Median(0)	Average
BP	Precision	35.65±0.67	**41.14±0.76**
	Recall	36.07±0.68	**41.45±0.76**
	F1-Score	35.84±0.67	**41.28±0.76**
CC	Precision	**41.55±0.93**	**41.34±0.97**
	Recall	**42.06±0.95**	**41.85±0.98**
	F1-Score	**41.77±0.94**	**41.56±0.97**
MF	Precision	47.02±0.54	**58.92±0.60**
	Recall	47.41±0.54	**59.47±0.60**
	F1-Score	47.18±0.54	**59.14±0.60**

Table S14: Performance of predicting noisy annotations on different τ in *S. cerevisiae* on archived GOA files (May, 2016). The numbers in **boldface** denote the best performance.

		Median(0)	Average
BP	Precision	40.39±0.61	**65.15±0.76**
	Recall	41.26±0.62	**65.94±0.76**
	F1-Score	40.78±0.61	**65.50±0.76**
CC	Precision	44.99±1.04	**57.90±1.20**
	Recall	46.36±1.07	**59.23±1.23**
	F1-Score	45.59±1.05	**58.48±1.21**
MF	Precision	44.89±0.55	**74.60±0.69**
	Recall	45.38±0.56	**74.80±0.69**
	F1-Score	45.08±0.55	**74.69±0.69**

Table S15: Performance of predicting noisy annotations on different τ in *A. thaliana* on archived GOA files (May, 2015). The numbers in **boldface** denote the best performance.

		Median(0)	Average
BP	Precision	**39.56±1.14**	**39.39±1.09**
	Recall	**40.08±1.16**	**39.91±1.11**
	F1-Score	**39.79±1.15**	**39.62±1.10**
CC	Precision	**73.39±3.54**	**71.51±3.58**
	Recall	**74.05±3.57**	**72.20±3.60**
	F1-Score	**73.66±3.55**	**71.80±3.59**
MF	Precision	**57.75±0.98**	52.53±0.96
	Recall	**57.96±0.98**	52.67±0.96
	F1-Score	**57.84±0.98**	52.59±0.96

Table S16: Performance of predicting noisy annotations on different τ in *H. sapiens* on archived GOA files (May, 2015). The numbers in **boldface** denote the best performance.

		Median(0)	Average
BP	Precision	33.62±0.54	**42.74±0.64**
	Recall	34.21±0.55	**43.45±0.65**
	F1-Score	33.87±0.55	**43.04±0.64**
CC	Precision	46.21±1.06	**61.77±1.34**
	Recall	46.93±1.07	**62.23±1.35**
	F1-Score	46.52±1.06	**61.98±1.35**
MF	Precision	45.27±0.89	**64.46±1.22**
	Recall	45.63±0.89	**64.77±1.22**
	F1-Score	45.42±0.89	**64.59±1.22**

Table S17: Performance of predicting noisy annotations on different τ in *S. cerevisiae* on archived GOA files (May, 2016). The numbers in **boldface** denote the best performance.

		Median(0)	Average
BP	Precision	**34.01±1.67**	**34.14±1.68**
	Recall	**34.22±1.68**	**34.34±1.69**
	F1-Score	**34.11±1.67**	**34.23±1.69**
CC	Precision	52.61±5.47	**59.77±6.28**
	Recall	52.99±5.51	**59.98±6.30**
	F1-Score	52.79±5.49	**59.87±6.29**
MF	Precision	35.19±2.07	**69.22±3.24**
	Recall	35.71±2.09	**69.82±3.25**
	F1-Score	35.38±2.08	**69.44±3.24**

4 Evaluation metrics for gene function prediction

Here, we introduce six evaluation metrics, *MacroAvgF1*, *MicroAvgF1*, *AvgAUC*, *AvgPrec Fmax* and *Smin* to measure the performance of gene function prediction. In fact, the first four metrics are widely to measure the performance of multi-label classification (Zhang *et al.*, 2014), which targets at predicting a set of related labels to an instance instead of a single label. From this perspective, gene function prediction can also be viewed as a multi-label learning problem. Therefore, these four metrics are used to measure the performance of gene function prediction (Fu *et al.*, 2016; Yu *et al.*, 2015). The latter two metrics (*Smin* and *Fmax*), along with AvgAUC, are suggested and used in the Second Critical Assessment of protein Function Annotation algorithms (CAFA2) (Jiang *et al.*, 2016). The definitions of these metrics are detailed in (Zhang *et al.*, 2014; Jiang *et al.*, 2016). To be self-inclusive, we list the definitions of these metrics here.

MacroAvgF1 is the average $F1$ scores of different terms:

$$MacroAvgF1 = \frac{1}{|\mathcal{T}|}\sum_{t\in\mathcal{T}}\frac{2p_t r_t}{p_t + r_t}$$

where $|\mathcal{T}|$ is the number of terms, p_t and r_t are the precision and recall for GO term t, defined as:

$$p_t = \frac{TP_t}{TP_t + FP_t} \quad r_t = \frac{TP_t}{TP_t + FN_t}$$

TP_t, FP_t, and FN_t are the true positive, false positive, and false negative with respect to term t. From the definition, it can be observed MacroAvgF1 first calculates the F1 measures for each term, and then averages over all the terms. MacroAvgF1 is more affected by the performance on the specific terms, which annotated to few genes.

MicroAvgF1 calculates the $F1$ measure on the predictions of different terms as a whole:

$$MicroAvgF1 = \frac{\sum_{t=1}^{|\mathcal{T}|} 2p_t r_t}{\sum_{t=1}^{|\mathcal{T}|} p_t + r_t}$$

MicroAvgF1 is more affected by the performance on general terms than on specific terms.

Average Precision (AvgPrec) evaluates the average fraction of terms ranked above a particular term $t \in \mathcal{T}_i$ ($\mathcal{T}_i$ stores all the GO terms annotated to the i-th gene).

$$AvgPrec = \frac{1}{N}\sum_{i=1}^{N}\frac{1}{|\mathcal{T}_i|}\sum_{t_1 \in \mathcal{T}_i} \frac{\{t_2 \in \mathcal{T}_i | rank(V(i,t_2)) \leq rank(V(i,t_1))\}}{rank(V(i,t_1))}$$

where $rank(V(i,\cdot))$ is a rank function, which ranks the largest $V(i,\cdot) \in \mathbb{R}^{|\mathcal{T}|}$ as 1 and the smallest $V(i,\cdot)$ as $|\mathcal{T}|$. The larger the value of AvgPrec, the better the performance is.

Average AUC (AvgAUC) is a term-centric evaluation metric, it averages the area under the receiver operating curve (AUC) of each term across all terms in $\mathcal{T}$. The receiver operating curve plots the true positive rate (sensitivity) as a function of the false positive rate (1-specificity) under different classification thresholds. The curve measures the overall quality of the ranking induced by the classifier, instead of the quality of a single value of the threshold in that ranking.

Fmax is a gene-centric evaluation metric used in the community-based critical assessment of protein function annotation (CAFA) (Radivojac *et al.*, 2013), Fmax is an F-measure computed as:

$$Fmax = \max_{\theta} \frac{2p(\theta)r(\theta)}{p(\theta) + r(\theta)}$$

where $p(\theta) = \frac{1}{m(\theta)}\sum_{i=1}^{m(\theta)} p_i(\theta)$ is the the precision at threshold $\theta \in [0,1]$, $p_i(\theta)$ is the precision on the i-th gene, $m(\theta)$ is the number of genes on which at least one prediction was made above the threshold θ, $r(\theta) = \frac{1}{N}\sum_{i=1}^{N} r_i(\theta)$ is the recall across N genes at threshold θ.

Smin is an evaluation metric used in the second critical assessment of functional annotation (CAFA2) (Jiang *et al.*, 2016) and defined as:

$$Smin = \min_{\theta} \sqrt{ru(\theta)^2 + mi(\theta)^2}$$

where the remaining uncertainty $ru(\theta) = \frac{1}{N}\sum_{i=1}^{N}\sum_{t} ic(t)\cdot \mathbb{1}(t \notin \mathcal{P}_i(\theta) \wedge t \in \mathcal{T}_i)$, misinformation $mi(\theta) = \frac{1}{N}\sum_{i=1}^{N}\sum_{f} ic(t)\cdot \mathbb{1}(f \in \mathcal{P}_i(\theta) \wedge t \notin \mathcal{T}_i)$. $\mathcal{P}_i(\theta)$ denotes the set of terms whose predicted scores greater than or equal to θ for the i-th gene, $\mathcal{T}_i$ denotes the ground-truth set of terms for that gene, $\mathbb{1}(\cdot)$ is an indicator function, and $ic(t)$ is the information content of the ontology term t. It is estimated in a maximum likelihood manner as the negative binary logarithm of the conditional probability that the term t is present in a gene's annotations given that all its parent terms are also present.

5 Removing noisy annotations improves gene function prediction

In the main text, we provide the experimental results of gene function prediction by a majority vote model on PPI networks with/without removing noisy annotations in archived GOA file of *H. sapiens*. Here, we report the corresponding results on archived GOA files of *A.thaliana* and *S. cerevisiae* in Tables S18-S19. From these tables, we can clearly see that removing the noisy annotations provides improved performance for gene function prediction.

Table S18: Results of gene function prediction on *A.thaliana* (archived date: May, 2016). The data in **boldface** denote the better result. 'Original' directly uses annotations in the historical GOA file to predict gene function; 'NoGOA' removes predicted noisy annotations from the historical GOA file and then predicts gene function. ↓ means the lower the value, the better the performance is.

	BP		CC		MF	
	Original	NoGOA	Original	NoGOA	Original	NoGOA
MicroAvgF1	82.10	**82.15**	**89.28**	**89.28**	85.65	**85.66**
MacroAvgF1	**74.43**	74.11	81.59	**81.60**	74.24	**74.61**
AvgPrec	76.70	**76.73**	**88.22**	**88.22**	82.77	**82.79**
AvgROC	**91.40**	91.03	**93.22**	**93.22**	91.72	**92.11**
Fmax	85.79	**85.80**	**92.27**	**92.27**	89.80	**89.84**
Smin ↓	8.08	**7.98**	**1.66**	**1.66**	2.78	**2.75**

6 Examples of predicted noisy annotations

Tables S20-S25 count the number of direct noisy annotations, predicted noisy annotations, and correctly predicted direct noisy annotations on *H. sapiens*, *A.thaliana* and *S. cerevisiae* archived in different years. Tables S26-S27 lists some examples of correctly (or wrongly) predicted direct noisy annotations.

Table S19: Results of gene function prediction on *S. cerevisiae* (archived date: May, 2016). The data in **boldface** denote the better result. 'Original' directly uses annotations in the historical GOA file to predict gene function; 'NoGOA' removes predicted noisy annotations from the historical GOA file and then predicts gene function. $\downarrow$ means the lower the value, the better the performance is.

	BP		CC		MF	
	Original	NoGOA	Original	NoGOA	Original	NoGOA
MicroAvgF1	94.60	**94.68**	94.74	**94.77**	95.36	**95.39**
MacroAvgF1	**90.24**	90.19	**92.28**	92.19	**93.36**	93.35
AvgPrec	92.26	**92.31**	94.99	**95.02**	94.87	**94.89**
AvgROC	**95.60**	95.24	**95.71**	95.42	97.53	**97.54**
Fmax	95.19	**95.20**	94.91	**94.92**	**96.21**	**96.21**
Smin $\downarrow$	1.52	**1.47**	**0.99**	**0.99**	0.60	**0.58**

Table S20: Statistics of predicted noisy annotations for different evidence codes on *A.thaliana* (archived date: May, 2016). $\bar{N}_c$ is the number of noisy annotations, $\hat{N}_c$ is the number of predicted and $\check{N}_c$ is the number of correctly predicted noisy annotations by NoGOA, respectively.

		TAS	EXP	IDA	IPI	IMP	IGI	IEP	ISA	ISO	ISM	RCA	IGC	IBA	IBD	IKR	IRD	ISS	NAS	IC	IEA
BP	$\bar{N}_c$	12	0	62	0	245	23	122	0	0	0	0	0	54	0	0	0	169	1	0	1003
	$\hat{N}_c$	21	0	66	0	275	24	103	0	0	0	0	0	219	0	0	0	166	2	0	1358
	$\check{N}_c$	10	0	38	0	178	16	54	0	0	0	0	0	53	0	0	0	137	1	0	927
CC	$\bar{N}_c$	3	0	332	2	1	0	0	0	0	0	0	0	33	0	0	0	72	4	0	339
	$\hat{N}_c$	8	0	398	4	1	0	0	0	0	0	0	0	84	0	0	0	112	5	0	388
	$\check{N}_c$	3	0	281	1	1	0	0	0	0	0	0	0	33	0	0	0	70	4	0	277
MF	$\bar{N}_c$	3	0	77	57	13	2	0	0	0	0	1	0	46	0	0	0	155	2	0	353
	$\hat{N}_c$	40	0	133	84	10	2	0	0	0	0	0	0	125	0	0	0	106	0	0	464
	$\check{N}_c$	3	0	72	53	8	1	0	0	0	0	0	0	43	0	0	0	102	0	0	333

Table S21: Statistics of predicted noisy annotations for different evidence codes on *H. sapiens* (archived date: May, 2016). $\bar{N}_c$ is the number of noisy annotations, $\hat{N}_c$ is the number of predicted and $\check{N}_c$ is the number of correctly predicted noisy annotations by NoGOA, respectively.

		TAS	EXP	IDA	IPI	IMP	IGI	IEP	ISA	ISO	ISM	RCA	IGC	IBA	IBD	IKR	IRD	ISS	NAS	IC	IEA
BP	$\bar{N}_c$	30	0	5	0	197	123	0	0	0	0	0	0	14	0	0	0	1	2	0	2743
	$\hat{N}_c$	742	0	37	0	105	50	2	0	0	0	0	0	165	0	0	0	27	11	1	4008
	$\check{N}_c$	22	0	2	0	81	49	0	0	0	0	0	0	12	0	0	0	0	0	0	2451
CC	$\bar{N}_c$	7	0	2	0	0	0	0	0	0	0	0	0	6	0	0	0	0	1	0	878
	$\hat{N}_c$	105	0	263	2	8	2	0	0	0	0	0	0	42	0	0	0	66	14	7	944
	$\check{N}_c$	6	0	2	0	0	0	0	0	0	0	0	0	6	0	0	0	0	0	0	660
MF	$\bar{N}_c$	9	0	2	1772	0	0	0	0	0	0	0	0	0	0	0	0	1	0	0	660
	$\hat{N}_c$	5	32	3	1401	0	1	0	0	0	0	0	0	164	0	0	0	0	3	0	1141
	$\check{N}_c$	3	0	2	1262	0	0	0	0	0	0	0	0	0	0	0	0	0	0	0	575

Table S22: Statistics of predicted noisy annotations for different evidence codes on *S. cerevisiae* (archived date: May, 2016). $\bar{N}_c$ is the number of noisy annotations, $\hat{N}_c$ is the number of predicted and $\check{N}_c$ is the number of correctly predicted noisy annotations by NoGOA, respectively.

		TAS	EXP	IDA	IPI	IMP	IGI	IEP	ISA	ISO	ISM	RCA	IGC	IBA	IBD	IKR	IRD	ISS	NAS	IC	IEA
BP	$\bar{N}_c$	121	0	94	5	222	62	5	0	0	0	0	0	406	0	0	0	9	5	0	829
	$\hat{N}_c$	147	0	95	19	331	81	5	1	0	1	0	0	516	0	0	0	33	4	0	1079
	$\check{N}_c$	112	0	66	2	176	49	5	0	0	0	0	0	392	0	0	0	9	2	0	731
CC	$\bar{N}_c$	149	0	114	7	6	0	0	0	0	0	0	0	285	0	0	0	1	2	12	149
	$\hat{N}_c$	102	0	269	38	19	0	0	0	0	0	0	0	338	0	0	0	3	4	7	136
	$\check{N}_c$	93	0	95	7	6	0	0	0	0	0	0	0	283	0	0	0	0	2	7	91
MF	$\bar{N}_c$	6	6	102	1513	27	9	0	0	0	0	0	0	259	0	0	0	20	1	0	69
	$\hat{N}_c$	6	4	130	1464	47	15	0	0	0	1	0	0	368	0	0	0	23	2	7	82
	$\check{N}_c$	5	4	85	1363	21	8	0	0	0	0	0	0	257	0	0	0	16	0	0	49

Table S23: Statistics of predicted noisy annotations for different evidence codes on *A. thaliana* (archived date: May, 2015). $\bar{N}_c$ is the number of noisy annotations, $\hat{N}_c$ is the number of predicted and $\check{N}_c$ is the number of correctly predicted noisy annotations by NoGOA, respectively.

		TAS	EXP	IDA	IPI	IMP	IGI	IEP	ISA	ISO	ISM	RCA	IGC	IBA	IBD	IKR	IRD	ISS	NAS	IC	IEA
BP	$\bar{N}_c$	0	0	18	0	1	1	0	0	0	27	18	0	57	0	0	0	5	0	0	339
	$\hat{N}_c$	2	0	22	0	40	16	11	0	0	27	85	0	97	0	0	0	9	0	0	323
	$\check{N}_c$	0	0	10	0	1	1	0	0	0	27	12	0	13	0	0	0	3	0	0	201
CC	$\bar{N}_c$	0	0	1	0	0	0	0	0	0	0	0	0	10	0	0	0	1	0	0	73
	$\hat{N}_c$	1	0	19	0	0	0	0	0	0	0	0	0	13	0	0	0	1	0	0	76
	$\check{N}_c$	0	0	1	0	0	0	0	0	0	0	0	0	10	0	0	0	1	0	0	68
MF	$\bar{N}_c$	0	0	0	1	0	0	0	0	0	0	0	0	2	0	0	0	0	0	0	805
	$\hat{N}_c$	9	0	80	52	11	3	0	1	0	0	0	0	27	0	0	0	52	1	0	837
	$\check{N}_c$	0	0	0	1	0	0	0	0	0	0	0	0	2	0	0	0	0	0	0	614

References

Fu,G. *et al.* (2016) NegGOA: negative GO annotations selection using ontology structure. *Bioinformatics*, **32**(19): 2996-3004.

Radivojac, P. *et al.* (2013) A large-scale evaluation of computational protein function prediction. *Nature Methods*, 10(3), 221–229.

Jiang,Y. *et al.* (2016) An expanded evaluation of protein function prediction methods shows an improvement in accuracy. *Genome Biology*, **17**(1), 184.

Table S24: Statistics of noisy annotations prediction for different evidence codes on *H. sapiens* (archived date: May, 2015). $\bar{N}_c$ is the number of noisy annotations, $\hat{N}_c$ is the number of predicted and $\check{N}_c$ is the number of correctly predicted noisy annotations by NoGOA, respectively.

		TAS	EXP	IDA	IPI	IMP	IGI	IEP	ISA	ISO	ISM	RCA	IGC	IBA	IBD	IKR	IRD	ISS	NAS	IC	IEA
BP	$\bar{N}_c$	125	3	35	0	13	0	0	0	0	0	0	0	75	0	0	0	7	22	0	2951
	$\hat{N}_c$	989	0	10	0	10	21	0	0	0	0	0	0	253	0	0	0	9	2	0	4087
	$\check{N}_c$	62	0	4	0	2	0	0	0	0	0	0	0	42	0	0	0	4	1	0	2588
CC	$\bar{N}_c$	2	0	8	0	5	0	0	0	0	0	0	0	11	0	0	0	6	0	0	733
	$\hat{N}_c$	4	0	24	0	2	0	0	0	0	0	0	0	42	0	0	0	6	0	0	948
	$\check{N}_c$	0	0	3	0	2	0	0	0	0	0	0	0	11	0	0	0	3	0	0	657
MF	$\bar{N}_c$	11	0	11	1	0	0	0	0	0	0	0	0	26	0	0	0	0	1	0	796
	$\hat{N}_c$	4	0	10	4	0	1	0	0	0	0	0	0	77	0	0	0	1	2	0	977
	$\check{N}_c$	4	0	5	1	0	0	0	0	0	0	0	0	24	0	0	0	0	1	0	713

Table S25: Statistics of noisy annotations prediction for different evidence codes on *S. cerevisiae* (archived date: May, 2015). $\bar{N}_c$ is the number of noisy annotations, $\hat{N}_c$ is the number of predicted and $\check{N}_c$ is the number of correctly predicted noisy annotations by NoGOA, respectively.

		TAS	EXP	IDA	IPI	IMP	IGI	IEP	ISA	ISO	ISM	RCA	IGC	IBA	IBD	IKR	IRD	ISS	NAS	IC	IEA
BP	N_c	13	0	25	0	20	9	0	0	0	0	0	0	0	0	0	0	17	0	6	82
	$\tilde{N}_c$	23	0	21	10	95	41	0	0	0	0	0	0	0	0	0	0	15	0	7	79
	$\check{N}_c$	9	0	7	0	9	4	0	0	0	0	0	0	0	0	0	0	8	0	6	52
CC	N_c	5	0	0	0	8	0	0	0	0	0	0	0	0	0	0	0	0	0	0	10
	$\tilde{N}_c$	6	0	6	3	5	0	0	0	0	0	0	0	0	0	0	0	0	0	0	10
	$\check{N}_c$	5	0	0	0	5	0	0	0	0	0	0	0	0	0	0	0	0	0	0	9
MF	N_c	4	0	0	3	2	0	0	0	0	0	0	0	0	0	0	0	0	0	0	96
	$\tilde{N}_c$	7	0	0	0	1	0	0	0	0	0	0	0	0	0	0	0	0	0	0	110
	$\check{N}_c$	4	0	0	0	1	0	0	0	0	0	0	0	0	0	0	0	0	0	0	88

Table S26: Examples of correctly ($\surd$) and wrongly($\times$) predicted direct noisy annotations by NoGOA in in MF branch of *S. cerevisiae.*

Protein		GO term	Evidence Codes	Details
AAC1(ADP/ATP carrier)	$\surd$	GO:0003735(structural constituent of ribosome)	IBA	GO_REF:0000033
AAC3(ADP/ATP carrier)	$\surd$	GO:0003735(structural constituent of ribosome)	IBA	GO_REF:0000033
AAD14 (Putative aryl-alcohol dehydrogenase AAD14)	$\surd$	GO:0005515(protein binding)	IPI	PMID:11805826
AAP1 (Alanine/arginine aminopeptidase)	$\surd$	GO:0005515(protein binding)	IPI	PMID:11805826
	$\times$	GO:0008270(zinc ion binding)	IBA	GO_REF:0000033
		GO:0042277(peptide binding)	IBA	GO_REF:0000033
		GO:0070006(metalloaminopeptidase activity)	IBA	GO_REF:0000033

Table S27: Examples of correctly ($\surd$) and wrongly($\times$) predicted direct noisy annotations by NoGOA in in BP branch of *S. cerevisiae.*

Protein		GO term	Evidence Codes	Details
AAC1(ADP/ATP carrier)	$\surd$	GO:0006412(translation)	IBA	GO_REF:0000033
AAC3(ADP/ATP carrier)	$\surd$	GO:0006412(translation)	IBA	GO_REF:0000033
AAP1 (Alanine/arginine aminopeptidase)	$\surd$	GO:0006267(pre-replicative complex assembly involved in nuclear cell cycle DNA replication)	IDA	PMID:16824194
		GO:0006270(DNA replication initiation)	IMP	PMID:17053779
		GO:0030466(chromatin silencing at silent mating-type cassette)	IDA	PMID:12897051
	$\times$	GO:0005977(glycogen metabolic process)	IMP	PMID:8100228
		GO:0006508(proteolysis)	IMP	PMID:8100228
		GO:0043171(peptide catabolic process)	IBA	GO_REF:0000033

Yu,G. *et al.* (2015) Predicting protein function via downward random walks on a gene ontology. *BMC Bioinformatics*, **15**, 271.

Zhang, M.L. *et al.* (2014) A review on multi-label learning algorithms. *IEEE Trans. Knowledge & Data Engineering*, 99, 1–1.
